# Programmes for people who are homeless and have severe mental illness in low-income and middle-income countries: a systematic review

**DOI:** 10.1016/S2215-0366(25)00206-8

**Published:** 2025-09-16

**Authors:** Lauren McPhail, Caroline Smartt, Christine Musyimi, Joel Agorinya, Sewit Timothewos, Fetuma Feyera, Ruth Tsigebrhan, Tigist Eshetu, Eleni Misganaw, Laura Asher, Ursula Read, Victoria Mutiso, David Ndetei, Charlotte Hanlon

**Affiliations:** Centre for Global Mental Health, Health Service and Population Research Department, Institute of Psychiatry, Psychology & Neuroscience, King’s College London, London, UK; Institute of Neuroscience and Cardiovascular Research, University of Edinburgh, UK; Africa Institute of Mental and Brain Health, Nairobi, Kenya; Department of Psychiatry, University of Oxford, Oxford, UK; Accra Psychiatric Hospital, Accra, Ghana; Centre for Innovative Drug Development and Therapeutic Trials for Africa, College of Health Sciences, Addis Ababa University, Addis Ababa, Ethiopia; Department of Psychiatry, Ambo University College of Medicine and Health Science, Ambo, Ethiopia; Department of Psychiatry, School of Medicine, College of Health Sciences, Addis Ababa University, Addis Ababa, Ethiopia; University of Rwanda, College of Medicine and Health Sciences, School of Medicine and Pharmacy, department of Psychiatry, King Faisal Foundation, Rwanda; Centre for Innovative Drug Development and Therapeutic Trials for Africa, College of Health Sciences, Addis Ababa University, Addis Ababa, Ethiopia; Mental Health Service Users Association–Ethiopia, Addis Ababa, Ethiopia; Nottingham Centre for Public Health and Epidemiology, School of Medicine, University of Nottingham, Nottingham, UK; Institute of Mental Health, University of Nottingham, Nottingham, UK; School of Health and Social Care, University of Essex, Colchester, UK; Africa Institute of Mental and Brain Health, Nairobi, Kenya; Department of Psychiatry, University of Nairobi, Nairobi, Kenya; Department of Psychiatry, University of Nairobi, Nairobi, Kenya; Institute of Neuroscience and Cardiovascular Research, University of Edinburgh, UK; Department of Psychiatry, School of Medicine, College of Health Sciences, Addis Ababa University, Addis Ababa, Ethiopia

## Abstract

Homelessness and severe mental illness are inter-related issues, the co-occurrence of which leads to poor outcomes for affected individuals. Evidence for effective interventions in high-income countries is accruing, but little is known about how to intervene in the diverse sociocultural contexts of low-income and middle-income countries (LMICs). The aim of this systematic review was to synthesise peer-reviewed and grey literature on programmes for people experiencing homelessness and severe mental illness in LMICs. We synthesised effects, programme components, and implementation strategies. We identified 80 sources describing 45 programmes across ten LMICs. Programme components spanned seven domains: service models, basic needs, health care, outreach, empowerment, community level, and macro level. Most programmes were multicomponent and included diverse delivery agents. Evaluation studies (n=21), although few in number and quality, reported clinical improvements; family reintegration ranged from 6% to 69%. Frequently reported implementation strategies included network weaving, educational meetings, and involvement of patients and family members. We identified programmes that show promise and can serve as starting points for local adaption. This systematic review identifies common domains of programmatic interventions that are important to include in combination for future programme design, while considering local contexts and population-specific needs. Future research should prioritise rigorous evaluations, with particular emphasis on programme effects and cost benefits.

## Introduction

Homelessness affects millions worldwide and is a focus for global efforts to achieve socially inclusive development (1). The number of people who are homeless in low-income and middle-income countries (LMICs) is probably underestimated and difficult to compare across countries due to differences in definitions, data collection methods, and incomplete geographical coverage (2). Homelessness has been defined as the absence of a fixed, regular, adequate nighttime residence (3); however, substantial cross-national differences in housing systems, social services, and cultural norms means that homelessness is operationalised differently across contexts (4). For example, estimates range from 100 million people who are homeless daily to more than 1 billion people living in precarious and inadequate housing (5). Increasing population pressure, rapid urbanisation, loss of land, economic hardship, and political instability all contribute to the growing phenomenon of homelessness in many LMICs (6). Policies of social control are prominent in responses to homelessness (7).

People with severe mental illness (defined here as mental illnesses associated with enduring disability, commonly psychotic disorders) are overrepresented in populations experiencing homelessness (5). A scoping review on homelessness and severe mental illness in LMICs found the estimated prevalence of severe mental illness in people who are sleeping on the streets or using temporary shelters varied from 8% to 47% (8). The relationship between homelessness and severe mental illness is complex and bidirectional, with cooccurrence leading to more adverse outcomes and a heightened risk of long-term homelessness (8). Outcomes are worse for particular groups, including women, who might experience gender-based violence (5). Substance use, either on its own or in the presence of severe mental illness, is a common comorbidity in people experiencing homelessness (9). Evidence from a rural Ethiopian setting showed that problematic substance use was synergistic in the exacerbation of symptoms of mental ill health and directly linked to loss of housing—for example, from not having money to pay rent for housing or from disagreements with other household members (8). Studies from both high-income countries (HICs) and LMICs have found increased duration of long-term home lessness in people who have comorbid substance misuse problems (10,11)

Current services globally are inadequate and often unavailable to meet the specific needs of people experiencing homelessness who have severe mental illness (PEHSMI); 80–100% of people who are homeless and have psychosis in Addis Ababa and Ethiopia reported multiple unmet health and social needs (12).

In many settings, programmes for PEHSMI focus on institutionalisation. Although institutionalisation can sometimes be a necessary step to initiate care and allow individuals to return safely to community living, it can involve human rights violations, abusive and coercive treatment, and involuntary hospitalisation (13). The UN Convention on the Rights of Persons with Disabilities advocates the right of people with psychosocial disabilities to live independently in their communities (14). Evidence for more community focused interventions for PEHSMI is accruing for HICs (15,16). Recommendations include integrating mental health specialties, such as psychiatric and addiction treatment, with other services to address unmet social and housing needs (2). More evidence from LMICs is needed so that interventions can be tailored to differing sociocultural contexts and respond to under resourced and fragmented mental healthcare systems, limited social welfare, and scarce housing options (1).

A scoping review of peer-reviewed publications from 1973 to 2018 found only one evaluated intervention for PEHSMI in LMICs, highlighting the need to search the grey literature (8). A systematic review of grey literature from LMICs that focused on community-based rehabilitation identified four programmes for people who are experiencing homelessness and have psychosocial disability (17). More investigation is needed to identify the full evidence base for programmes targeting PEHSMI in LMICs to determine what is feasible, acceptable, and beneficial in these contexts.

The aim of this systematic review was to synthesise evidence on programmes for PEHSMI in LMICs. Our research questions were designed to (1) identify programmes for PEHSMI that have been implemented or evaluated, or both, in LMIC settings and establish which implementation strategies have been used; and, (2) to establish the experience and evidence of the impact of such programmes at the individual, family, community, provider, or system level, or combinations thereof.

## Methods

This systematic review was conducted as part of the work of the National Institute of Health and Care Research (NIHR) group on homelessness and mental health in Ethiopia, Ghana, and Kenya (HOPE) (4). We adhered to the 2020 PRISMA guidelines (18). The protocol was registered on PROSPERO (CRD42023485339).

### Search strategy and selection criteria

We initially searched for studies published between Jan 1, 2003, and Dec 31, 2023 from our original search in 2023. Following updated searches in 2024, the period covered was 21 years. All languages were included. For records in languages other than English, we used Google Translate to translate abstracts and determine eligibility for full text screening. Studies were restricted to those conducted in LMICs based on World Bank classifications at the time of publication (19). Studies that met the criteria of individuals aged 16 years or older who were homeless and had severe mental illness were included. Homelessness was operationalised as current literal (street) home lessness; current residence in a homeless shelter, refuge, or hostel, or living illegally in uninhabited buildings; current admission in psychiatric facility, jail, or religious healing community, with an immediately preceding homeless episode and/or no place of residence to go post hospital discharge; and previous street homelessness. Severe mental illness was operationalised according to ICD10 and DSM5 (or earlier diagnostic systems where relevant—eg, DSMIV) criteria or clinical assessment indicating a primary psychotic or affective psychotic disorder (including bipolar disorder). In addition, we searched for studies using broader terms indicating severe mental illness, including psychosis, psychosocial disability, mental disability as operationalised by the implementing organisation, and also included terms for developmental disability, autism or dementia, with the expectation that relevant programmes in LMICs were unlikely to be focused narrowly on specific diagnoses (8). Studies including people with other mental health conditions (ie, posttraumatic stress disorder, substance use, obsessive compulsive disorder, and major depressive disorder without psychotic symptoms) were only included if outcomes were reported separately for people with severe mental illness or if people with severe mental illness accounted for more than 50% of the sample. We included qualitative, quantitative, and mixed methods evaluations and description studies that systematically reported on programmes, implementation, and participant experiences. We excluded studies involving people with substance use disorders in the absence of severe mental illness, studies from acute humanitarian settings, or studies focused on people considered at risk of homelessness, but without past or current experience of homelessness, or people living in substandard housing.

LM developed the search in Embase and then adapted it to MEDLINE, PsycINFO, Cochrane Central Register of Controlled Trials, Global Health, Cumulative Index to Nursing and Allied Health Literature, PsycARTICLES, Web of Science, and Global Index Medicus. For the grey literature, LM and CS searched PsycExtra, WorldCAT, Google Scholar, WHO Registry for Clinical Trials, ClinicalTrials.gov, the World Health Organisation Library, and the UN Human Rights Library. In addition, we manually searched relevant websites, lists of organisations and nongovernmental organisations (NGOs), and Google. LM constructed search terms for four different domains: homelessness, severe mental illness, low-income and middle-income countries, and interventions (appendix 1, 2). Manual searching by review raters (LM, CS, CM, JA, ST, FF, RT, and TE) consisted of forward and backward citation tracking of all included papers and related systematic reviews. LM and CS led the expert consultations over email, as well as an extensive snowballing process through people and organisations. Consortium members in Ethiopia, Ghana, and Kenya (CM, JA, ST, FF, RT, and TE) led the snowballing in their respective countries. Study reports were requested through email, by LM, from authors of conference abstracts.

Electronic searches conducted by LM and manual searches by review raters were rerun before the final analysis in June, 2024. LM exported records to EndNote 20 for deduplication, and then to Rayyan for screening. An initial subset of 100 records was screened independently by eight raters (LM, CS, CM, JA, ST, FF, RT, and TE) to establish consistency. Any disagreements were discussed and resolved. All titles and abstracts were independently screened by two raters with records equally split and allocated between the eight raters. For studies that progressed to full text screening, the same double blinded screening process was repeated with the same eight raters. Conflicts were resolved by consensus between the eight raters and CH.

### Data analysis and narrative synthesis

We designed and prepiloted a data-extraction form with the following template: study identification, country, author or authors, setting, population characteristics, intervention, implementation strategies, outcome measures, outcome data, and findings. Outcomes were not predetermined as primary or secondary. Two reviewers independently extracted data for each included article; articles were split equally between the eight raters. An independent rater (LM) then merged information from the two reviewers to check for any inconsistencies. Where there were discrepancies, data were cross-checked for accuracy and differences resolved through discussions with the review team (CM, JA, ST, FF, RT, TE, CS, LM, and CH). A mixed methods appraisal tool (MMAT) was used to assess the quality of any studies where the criteria could be applied (20). This assessment was done by the same two reviewers completing the data extraction separately, and collaborative discussions among the review team settled discordance. The total MMAT score indicated the overall quality of the appraised study, with 0% indicating low quality and 100% high quality. Studies were not excluded based on methodological quality.

To address review questions, we did a narrative synthesis following guidance by Popay and colleagues (21), structured around the type of programme. LM and CS synthesised evidence on programme components and implementation strategies; experience of care; and effect at the individual, family, community, and provider or system levels. Programmes were referenced by a code (appendix 4). Programme domains were developed based on a brief scoping search of the literature conducted before the main review and drew on emerging findings from a global expert consensus exercise and discussions within our research consortium (4). They were then iteratively improved in response to review findings. CH contributed to synthesising findings and data interpretation. CS mapped implementation strategies onto the Expert Recommendations for Implementing Change (ERIC) strategies (22). The narrative synthesis incorporated consideration of the quality appraisal assessment results. Comparative analyses were prespecified; however, the small number of studies reporting quantitative outcomes and the substantial heterogeneity of studies precluded a metanalytical approach.

## Results

**Figure 1:**
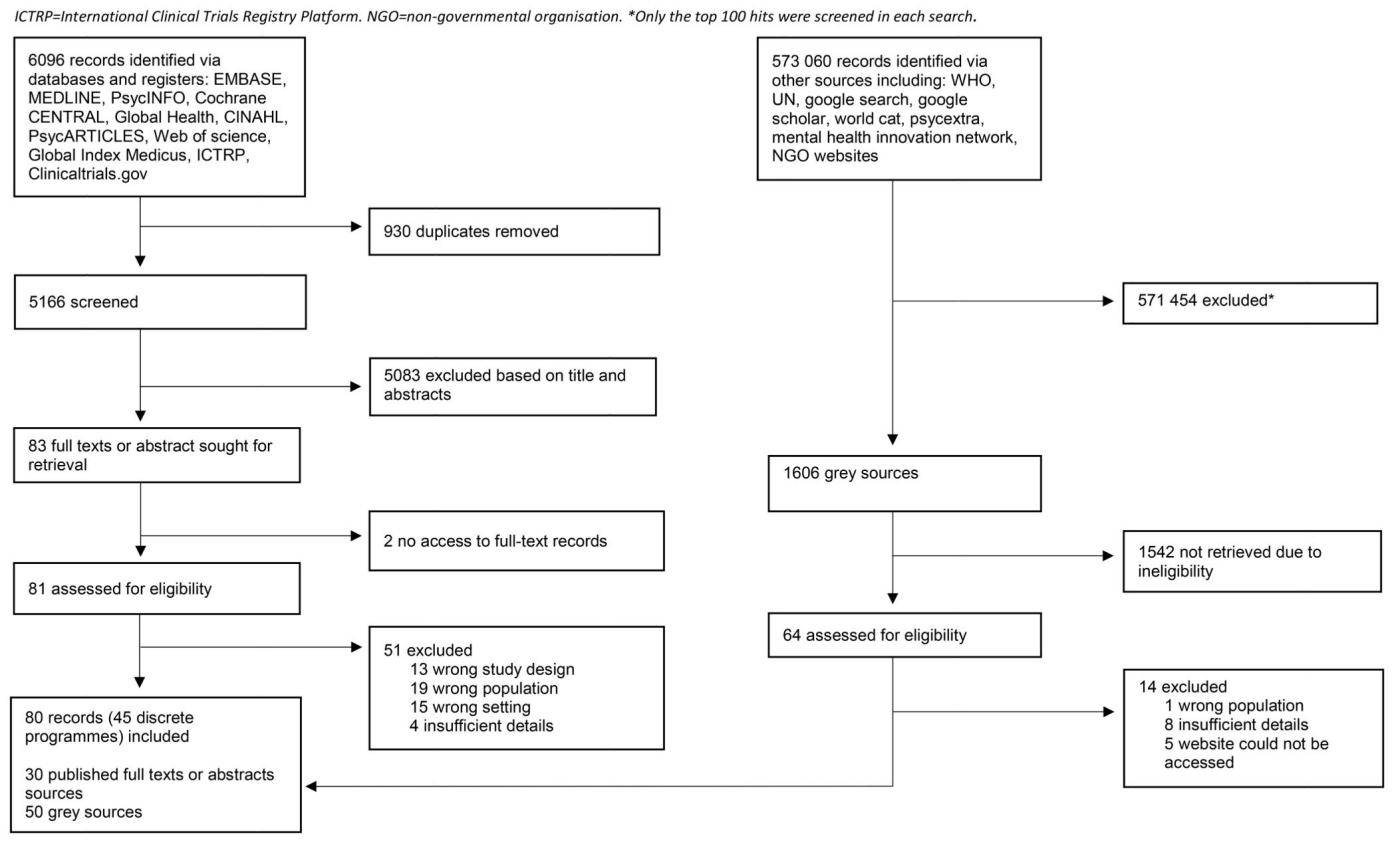
Study Selection

We identified 5166 unique records from the database searches for title and abstract screening, 81 of which were assessed for eligibility. We identified an additional 1606 sources from the grey literature, and 64 were assessed for eligibility. 20 experts were consulted. 80 sources, 30 peer reviewed and 50 grey literature, were included that related to 45 programmes (Figure 1).

The 30 peer-reviewed publications pertained to 16 programmes. These sources were either service descriptions (n=14) or evaluation studies (n=16). Publication dates ranged from 2006 to 2024. The overall quality of the 15 studies amenable to risk of bias assessment was mixed (appendix 5). The grey literature search identified 50 sources for 37 discrete programmes—eight already identified from the peer reviewed scientific literature—of which 34 sources were identified from websites, often featuring programme information, involvement opportunities, testimonies and annual or audit reports; five evaluations; four online news sources; three reports (ie, WHO and national government); three book chapters; and one draft state policy.

Programme characteristics are presented in table 1. The identified programme domains were basic needs, health care, outreach, service models of care, empowerment, community level, and macro level. Definitions and programme components for each domain are detailed in the appendix (appendix 6). ERIC implementation strategies are presented by code number (1–73) in the appendix (appendix 7).

Most programmes were from India (n=34). We identified one programme from each of Brazil, Liberia, Mozambique, Nigeria, Cameroon, Bangladesh, and Myanmar. A further two programmes were from Ghana, and one programme spanned Côte d’Ivoire, Benin, and Togo. Programmes were most commonly run by NGOs (n=37) or governmental organisations (n=8). A range of delivery agents were included within each programme, comprising lay workers (including NGOs, volunteers, community-based rehabilitation workers, civil society workers, and disability workers; n=26), mental health professionals (n=25), allied mental health professionals (n=17), general practitioners (n=14), police and justice system staff (n=13), general health professionals (n=11), community health workers (n=8), allied health professionals (n=9), peers (n=5), family members (n=2), and traditional or faith healers (n=2).

Most programmes were community based (n=38). Community-based rehabilitation principles were followed by Edawu, a mental health rehabilitation unit in rural Nigeria, as it shifted its focus from inpatient rehabilitation to intervention in the community (49). Residential or day rehabilitation centres were described in 27 programmes. Hospital inpatient services were offered by 15 programmes, with some admissions occurring under judicial orders or police custody. 15 programmes provided hospital outpatient services. One programme, Altruist, provided mental health services alongside traditional faith healing at religious places. Another emphasised the crucial role of intersectoral coordination and particularly social assistance services as entry points to the pathway to access care (45). Only six programmes provided integrated services for mental health and substance use.

Most programmes engaged with individuals on the streets (n=40) through various approaches. The Koshish outreach initiative reaches the most vulnerable on the streets during the night and connects people to the nearest shelter or hospital. In Brazil, their street outreach involves occupational therapists to help identify the needs of PEHSMI (45). The police were also commonly used in outreach efforts or programmes partnered with police to facilitate hospital admission through a legal framework (n=13). Some (n=2) specifically mentioned using police or local authorities to provide security for outreach teams. Iswar Sankalpa outreach provides mental health care and allied services on the streets through a multidisciplinary team, including caregivers who are identified from the community (61). With their services provided on the streets, individuals are not coerced into treatment and can withdraw at any time (34). Crisis interventions, including ambulances, paramedics, and helplines, were included in 18 programmes to link people for immediate mental health assessment and treatment.

All 45 programmes offered sanitation and washing facilities, food, clothing, and protection through shelter, residential centres, day rehabilitation centres, or hospital inpatient admission. Some programmes provided standalone support for basic needs—for example, monthly food rations in Liberia (70)—or a daily meals on wheels service directly to PEHSMI on the streets of Tamale, Ghana. Some programmes provided longer-term accommodation (n=14), with 11 modelled after the Banyan’s Home Again initiative, which provides community-based supported housing for PEHSMI, helping them to transition from institutional care to independent living with supportive services. Through Open Dialogue sessions and regular social visits, the Banyan’s Home Again programme actively addresses any concerns with coercion (34). A further five programmes provided transitional, time-limited facilities for those exiting hospital, night, or 24-h temporary shelters.

Psychiatric assessment and diagnosis (n=38) and provision of psychotropic medication (n=36) were a component of most programmes. Some (n=5) programmes linked with local general or psychiatric hospitals for specialist treatment. Others, such as Edawu, incorporated training of the WHO mental health Gap Action programme for nonspecialist health workers to provide evidence-based mental health care (49,50). Support with medication adherence (n=5) was less frequently described. Partners in Health Liberia described monitoring medication side effects and administration for all individuals during daily visits from community health workers (70). At Aung Clinic, mental health treatment is on a voluntary basis, with staff trained to use de-escalation measures to avoid coercion around medication or involuntary treatment or admissions (34). Psychological interventions were used in 28 programmes, 15 provided psychoeducation, and 26 included psychosocial interventions. The Aung Clinic provides art therapy, group therapy, and offers peer-based support groups (34). Physical health care was provided by 25 programmes. Occupational therapy within a mental health setting (n=16) and substance use interventions (n=4) were less frequent.

Self-help interventions were seen in 25 programmes, including life or social skills training and education, or both. Integration and inclusion programmes to encourage the participation of PEHSMI in the community were described in 18 programmes. Legal support was included in 12 programmes. Some programmes registered individuals under social welfare departments to obtain documentation for PEHSMI (n=8). Economic support was provided by 11 programmes, including waiver of hospital charges and travel costs. Livelihood opportunities were provided by 22 programmes. Vocational training was a component of 28 programmes, social work interventions were present in 11 programmes, and recreational therapy in 15 programmes. Support for participation in religious activities was identified in just six programmes.

Family integration, through tracing and reuniting individuals with families, was a common component in 40 programmes. Reintegration was described as an extensive process beginning with stabilising the individual’s condition, gathering family information and their home address, and attempting to identify unknown individuals (without identification documents). For some, information was crosschecked against online resources, databases, or biometric systems—particularly common for those admitted to hospital inpatient settings—and through networking with other organisations. If families were found and willing to take the individual home, family psychoeducation was usually provided. Ethical considerations when reaching out to the family members of PEHSMI were inconsistently addressed, with some programmes contacting family members before an individual could consent. Community integration and engagement efforts, such as establishing linkages with community stakeholders (n=31), were frequently included. Awareness raising about mental health and homelessness was included in several programmes (n=25). Stigma and discrimination reduction programmes in the community (n=7) and consultations with gate keepers and opinion leaders (n=2) were less common.

Programme components at the macro level were the least often identified. Programmes (n=25) established linkages between national, regional, and transnational structures, often referred to as public–private partner ships. Challenges between these partnerships were noted, with restrictions placed on NGOs to access foreign funding (63). The development of national policies (ie, strategic mental health plans) was seen in only three programmes.

The frequency of ERIC implementation strategies described by each individual programme ranged from one to 34 of a total of 73 discrete strategies detailed in the appendix (appendix 8). The most widely used ERIC implementation strategy was to promote network weaving (n=28). This result reflects the need for a multidisciplinary approach to meet varied needs and to use any existing available resources. Other identified ERIC strategies were to conduct educational meetings (n=23) to sensitise various stakeholders and involve them in the programme implementation; to involve patients and family members (n=15); to change service sites (n=13); to prepare patients to be active participants (n=10); to access new funding (n=10); to obtain and use patient and family feedback (n=9); and to use advisory boards and workgroups (n=9). These strategies were found in more than 20% of all programmes.

Studies that included an evaluation component of mixed low risk and high risk of bias are presented in table 2 and table 3. Studies using an evaluation method (n=21) included chart reviews (n=11), pre-evaluation and post evaluation (n=2), qualitative evaluation (n=2), mixed quantitative–qualitative methods (n=4), cross-sectional design (n=1), and matched group design (n=1). Sample sizes varied from N=9 to n=1114.

All studies with an evaluation component obtained data from people with lived experience. Quantitative outcomes were mostly reintegration and rehabilitation (n=12) or clinical (n=8), with fewer studies reporting employment (n=4), social (n=2), quality of life (n=2), cognitive (n=1), or basic needs (n=1) outcomes. Studies used standardised scales, including the Clinical Global Impression, WHO Quality of Life, and Rating on Periodic Psychiatric Assessment chart. Clinical outcomes showed notable symptom reduction, improved selfcare, and improved cognitive and psychosocial functioning. Medication assessment outcomes revealed 42% of individuals were receiving regular medication; however, gaps were seen in adherence or access. Reintegration emerged as a primary outcome, with family reintegration ranging from 6% to 69%. Employment outcomes were varied, with some programmes reporting 15–36% employment, although barriers such as skill loss and mental health relapse persisted. A study with high risk of bias investigating employment outcomes among ex-residents of Amaudo’s residential facility found only one third were engaged in work after 3 years (24). In a study in South India, following an initial decline after the first month of moving from a hospital setting to supported housing, all quality of life domains showed a steady upward trend during the follow-up assessments conducted at months 4, 5, and 6 (41).

Qualitative findings emphasised the importance of safe and inclusive living environments, the value of vocational training, and the positive effect of art and group therapy sessions—people attending the Aung Clinic noted finding acceptance and feeling more able to manage their conditions since attending (35). Some studies also discussed the challenges faced in implementing interventions, including difficulties in family tracing and reintegration. These challenges were associated with family reluctance due to financial, social, or knowledge barriers. Among individuals for whom reintegration was neither possible nor desirable, supported independent living options with bespoke housing supports led to substantial community integration gains. These gains were fostered through the development of a culture of family and home (41). Several studies emphasised the importance of multisectoral collaboration, particularly between health services, social services, and law enforcement, in effectively reaching and treating PEHSMI. Whereas most studies reported positive outcomes, they also highlighted ongoing challenges at various levels.

Three studies provided a cost analysis of the service provision per participant (monthly or daily). The funding for these three programmes was sourced from nonprofit organisation partners.

## Discussion

We identified 45 programmes for PEHSMI in LMICs. Most programmes used a multicomponent approach to address the needs of PEHSMI, combining individual-based, family-based, and community-based interventions. Hospital-based services and residential rehabilitation facilities were the most common programme models, enabling initial stabilisation of a person’s circumstances and response to basic needs. Diverse personnel were engaged in the delivery of programmes, broadly grouped into community-based (NGOs, volunteers, community-based rehabilitation workers, civil society workers, disability workers, community health workers, peers, family members, and traditional or faith healers) and those in formal services (mental health specialists and allied professionals, general health workers and allied health professionals, and the police and justice system).

Psychiatric treatment and medication were commonly reported programme components, reflecting their crucial role in the immediate management of severe mental illness among people experiencing homelessness. Less emphasised were physical health interventions, despite high excess mortality and morbidity in people with severe mental illness, expected to be even higher in population who are experiencing homelessness (1, 12). There was also little mention of addiction services, which warrants further investigation—particularly through a better understanding of relevant policy contexts. Six evaluation studies included participants with substance use disorder, providing integrated services, but did not contain descriptions of specific interventions to address addiction. Except for Amaudo, Association St Camille, Aung Clinic, The Banyan, and Iswar Sankalpa, reports were unclear about whether admissions to hospital or residential programmes were involuntary and did not report on adverse incidents within these settings, for example, restraint or other harms.

Family reintegration emerged as the primary long-term strategy for addressing homelessness, followed by time limited rehabilitation in residential facilities. Admissions into residential facilities, along with extended facility stays, probably indicate an absence of alternative accommodation and treatment options for PEHSMI in most LMICs. Nonetheless, we found emerging evidence for early success with independent supported housing for those who cannot live independently or for whom family reintegration is not possible or desirable (41). An ongoing clinical trial is testing the feasibility of a supportive housing programme on independent living skills of homeless women with severe mental illness in India (CTRI/2022/06/043323).

The complexity and extensive processes required to trace families and achieve reintegration underscores the need for dedicated resources and strategies to ensure successful outcomes. Such strategies include tracing families through national databases, telephone, postal letter, collaborating with organisations, home visits, and the media (67,69). Family-based components, in particular family psychoeducation, have been described as key to successful rehabilitation, given the salience of family support in recovery and to help strengthen social networks (26,27). Family psychoeducation is widely regarded in the literature as an important component of family based interventions for psychosis in LMICs, which can be used in diverse settings (78). Stigma, misconceptions, and little accessible and affordable treatment were identified as reasons for not initiating treatment and drivers of pathways into homelessness (65). Preconceived ideas pose significant barriers to acceptance and inclusion in the reintegration process (63). A history of interpersonal issues within families adds further complexity; solutions that work in one family might not yield the same results in another (68). Engaging family members in the process of treatment was one of the most used implementation strategies, with over half the identified programmes explicitly describing this process.

Establishing linkages and coordination among community stakeholders was frequently reported and nearly two-thirds of programmes clearly described promoting network weaving as key to implementation efforts. Collaboration across various sectors is needed for the successful reintegration and rehabilitation of PEHSMI, while aiming to ensure comprehensive care by addressing multifaceted needs. Establishing community based psychosocial rehabilitation centres collaboratively run between professionals, family, and community resources is a common initiative to enable recovery for people with severe mental illness in LMICs (15).

We identified several key community actors involved in outreach intervention efforts, including volunteers, allied health professionals, and police and ambulance personnel, all of whom play a role in facilitating access to treatment. Community-based approaches, including outreach, have the potential to protect human rights of PEHSMI (16). There is a need, however, to exercise caution when considering the role of the police in these programmes. A dichotomy emerged in outreach approaches: some programmes relied on the police for security, prioritising immediate safety concerns and reflecting an enforcement-focused approach, whereas others focused on engagement and approaches tailored to specific individual needs. This divergence might be a consequence of the limited availability of and low salaries for professionals in these contexts, which often results in a heavy reliance on volunteers (25). At the Shekhinah clinic, every member of staff is an unpaid volunteer—some specialised or otherwise unspecialised—who supports the clinic of their own free will.

Empowerment components, including livelihood opportunities, social work interventions, and vocational training for skill and competencies needed for employment were found in some programmes, but with insufficient evidence to assess their effectiveness. Consequently, there is a need to further investigate targeted job training, employment programmes, and linkages to livelihood opportunities; absence of employment opportunities is a structural factor that promotes and maintains homelessness (2,24). Fewer intervention components were described at the macro level. The absence of intervention components does not necessarily indicate a lack of effectiveness; instead, it might reflect the difficulties NGOs face in scaling up macrolevel components due to competing governmental priorities and structural barriers that result in few government welfare and poverty alleviation schemes, underfunding, and the absence of social housing (1). These challenges underscore the importance of stronger advocacy and policy development to address the needs of PEHSMI at a systemic level.

This systematic review is, to our knowledge, the first to synthesise all peer-reviewed and grey literature sources on interventions for PEHSMI in LMICs. The quality of evidence was mixed, with a paucity of evaluation studies and insufficiently detailed and systematic description of intervention components and implementation strategies. These programmes are currently insufficiently represented in scholarly work, and it is possible that we have not identified all sources for inclusion. Most implementation strategies were not systematically reported, nor were data sources intended for an implementation science audience, limiting the rigor of the comparison (79). The substantial grey literature included in this systematic review, largely drawn from websites often oriented towards funders or potential service users and their families, might not include implementation parlance. In practice, more strategies might be used than described here. Ethical considerations were inconsistently addressed or were not assessed in the literature. This systematic review focused on programmatic interventions and did not include broader policy-level or structural approaches, such as regional frameworks like the Southern African Development Community Protocol on Combating Illicit Drug Trafficking (80), which might also play an important role in addressing substance use and homelessness. Furthermore, our pragmatic inclusion criteria requiring outcomes for PEHSMI to be separately reported or to account for more than 50% of the sample might have inadvertently excluded relevant studies.

Despite a notable growth of research investigating interventions for PEHSMI within LMICs, the evidence base is still limited and geographically restricted, with most peer-reviewed evidence coming from India. We identified programmes that show promise and can serve as starting points for local adaptation; the Banyan, in particular, has evolved over three decades to integrate with existing systems and provide comprehensive, rights-based care (39). Our systematic review also identifies common domains of programmatic interventions that are important to include in combination for future programme design, while considering local contexts and population-specific needs. The diversity of personnel employed across programmes indicates the potential for flexibility depending on context.

Researchers should increase their efforts to investigate interventions for PEHSMI in LMICs. Use of checklists for describing complex interventions (ie, template for intervention description and replication) could improve replication and modelling of important intervention components (81). There is a need for rigorous evaluations of programme effects and cost–benefits; however, conventional randomised controlled trials might not be possible, informative, or ethical due to the multifaceted and complex nature of these programmes. Instead, stepped-wedge trial designs, implementation research, or realist evaluation might be better suited to obtaining policy-relevant evidence (82). Qualitative exploration of the experiences of programmes from the perspectives of service users, as well as barriers and facilitators to programme success, is needed.

## Conclusion

This systematic review assessed the use of multi-component approaches to support PEHSMI in LMICs. We have identified several programmes with promising models, featuring diverse delivery agents and a combination of components that can be adapted to meet local, context-specific needs. Future research ought to focus on rigorous evaluations of interventions, including qualitative studies, to understand barriers and facilitators from the perspective of those with lived experience. Strong local partnerships and integrated comprehensive care models are key to effectively address the diverse needs of this population, promote their inclusion and protect their rights.

## Supplementary Material

Supplementary Appendix

## Figures and Tables

**Table 1:** Characteristics of 45 discrete programmes

Programme	Primary service model for PEHSMI	Approach to addressing homelessness	Entry into programme	Delivery agents	Included programme domains	Peer-reviewed sources	Grey sources
Aashray Adhikar Abhiyan, India (23, 83)	Non-residential, community based, NGO	Reintegration, temporary shelter	Outreach teams	Lay workers	All domains	0	2
Altruist, India (84)	Non-residential, community based, NGO	Reintegration, long-term group homes	Outreach teams	Traditional or faith healers, lay workers	All domains	0	1
Amaudo Itumbauzo, Nigeria (24–27, 85)	Residential centre, NGO	Residential shelter, reintegration	Outreach teams	MHPs, GHPs, CHWs, lay workers	All domains	3	2
Anbagam, India (86)	Residential centre, NGO	Residential shelter, reintegration	Outreach teams	MHPs, MHP allied, GPs, GHP allied, GHPs	All domains except macro level	0	1
Apna Ghar Ashram, India (28, 87)	Residential centre, NGO	Residential shelter, reintegration	Outreach teams, dedicated ambulances	MHP allied, GPs, GHPs, lay workers, police and justice system, GHP allied	All domains	0	2
Ashadeep, India (29–32, 34, 88)	Residential centre, NGO	Residential shelter, reintegration, long-term group homes	Outreach teams	MHPs, MHP allied, CHWs, GHPs	All domains	0	6
Association St Camillie de Lellis, Côte D’Ivoire, Benin and Togo (25, 33, 89)	Residential centre, NGO	Residential shelter, reintegration	Outreach teams	MHP, GHP, CHW, lay workers, peers	All domains except macro level	2	1
Atchayam Trust, India (90)	Residential centre, NGO	Residential shelter, reintegration	Outreach teams	Lay workers, GPs, CHWs, GHPs	All domains	0	1
Aung Clinic, Myanmar (34,35, 91)	Non-residential rehabilitation day centre, NGO	Temporary shelter	Outreach day services only	MHPs, GPs, peers	All domains except macro level	0	3
The Banyan, India (28, 29, 31, 34, 36–42, 43, 44, 92)	Residential centre, NGO	Residential shelter, reintegration, long-term group homes	Outreach teams, conveyed by police or volunteers	MHPs, MHP allied, GPs, GHPs, GHP allied, CHWs, lay workers, police and justice system, peers	All domains	6	8
BasicNeeds, Ghana (93)	Non-residential community-based, NGO	Reintegration is first-line strategy, alternatively, PEHSMI are taken to other local resources (Shekinah clinic)	Outreach teams	Lay workers, peers	All domains, except macro level	0	1
CAPS unit (Psychosocial Community Centres) and night shelter, Brazil (34,35)	Intersectoral night shelter and social assistance, governmental	Temporary shelter	Outreach teams	MHP allied, lay workers, GHPs	All domains except community level and macro level	1	1
Chittadhama, India (28, 46–48)	Residential centre, NGO	Residential shelter, reintegration	Conveyed by police	MHPs, MHP allied, police and justice system	All domains	0	4
Edawu, Nigeria (50–53)	Residential centre, NGO	Residential shelter, reintegration	Outreach teams	MHPs, CHWs, lay workers	All domains, except macro level	1	4
Government Medical College and Hospital, India (54)	Hospital inpatient, governmental	Institutional, reintegration	Conveyed by police	MHPs, MHP allied, police and justice system	All domains, except outreach	1	0
Gramin Advasi Samaj Vikas Sansthan, India (94)	Residential centre, NGO	Residential shelter, reintegration, long-term group homes	Outreach teams	Not stated	All domains	0	1
Green Dot Trust, India (95)	Residential centre, NGO	Residential shelter, reintegration, long-term group homes	Outreach teams	MHPs, MHP allied, lay workers	All domains	0	1
Gujarat Government psychiatric care facilities, India (55)	Hospital inpatient, governmental	Institutional, reintegration	Conveyed by police or volunteers	MHPs, police and justice system	All domains, except outreach	1	0
INCENSE (Parivartan Trust), India (28, 31, 56, 57)	Non-residential community-based, NGO	Reintegration	Outreach teams, transition from institutional	GPs, MHP allied, peers, lay workers, family members	All domains	0	4
Indian Legislation in rehabilitation, India (58)	Hospital inpatient, governmental	Institutional, reintegration	Through the Mental Healthcare Act, 2017, with a specific role for the police	MHPs, lay workers, police and justice system	All domains, except macro level	1	0
Infulene Psychiatric Hospital, Mozambique (59)	Hospital inpatient, governmental	Institutional, reintegration	Mapping and recruitment through involvement with community stakeholders	MHPs, MHP allied, lay workers	All domains, except macro level	1	0
Iswar Sankalpa, India (30, 60–64)	Residential centre, NGO	Residential shelter, reintegration	Outreach teams, conveyed by police or volunteers	MHPs, MHP allied, GPs, GHPs, GHP allied, CHWs, lay workers, police and justice system, peers, family members	All domains	2	4
Jewels international, India (97)	Non-residential community-based, NGO	Long-term group homes	Not specified	Not stated	All domains	0	1
King George Medical University, India (26, 65, 77)	Hospital inpatient, governmental	Institutional, reintegration	Admissions through valid legal orders	MHPs, lay workers, police and justice system	All domains	2	1
Koshish, India (98)	Non-residential community-based, NGO	Temporary shelter	Outreach teams	Not stated	All domains	0	1
Karuna Trust, India (28, 99)	Residential centre, NGO	Residential shelter, reintegration	Outreach teams	MHP allied, GPs	All domains	0	2
La Village de L’amour, Cameroon (66)	Residential centre, NGO	Residential shelter, reintegration	Outreach teams	MHPs, GPs, GHP allied, lay workers	All domains, except macro level	0	1
Maher Ashram, India (57, 100)	Residential centre, NGO	Residential shelter, reintegration	Outreach teams	Not stated	All domains, except macro level	0	2
Mariyasadanam Charitable Trust, India (29, 101)	Residential centre, NGO	Residential shelter, reintegration, long-term group homes	Outreach teams	MHPs, GPs, GHP allied, traditional or faith healers, MHP allied	All domains, except macro level	0	2
Menadora Foundation, India (102)	Residential centre, NGO	Residential shelter, reintegration, long-term group homes	Not specified	MHPs, lay workers	All domains	0	1
MS Chellamuthu Trust & Research Foundation, India (103)	Residential centre, NGO	Residential shelter, reintegration	Outreach teams	Not stated	All domains	0	1
National Institute of Mental Health and Neurosciences, India (67–69)	Hospital inpatient, governmental	Institutional, reintegration	Admission under judicial reception order, conveyed by police	MHPs, MHP allied, lay workers, police and justice system	All domains, except empowerment and macro level	3	0
Paripurnata, India (104)	Residential centre, NGO	Residential shelter, reintegration	Transition from institutional	Not stated	All domains	0	1
Community-based mental health, Harper and Pleebo districts in Maryland County, Liberia (70)	Non-residential community-based, NGO	Reintegration, long-term group homes	Mapping and recruitment through involvement with community stakeholders	MHPs, CHWs, lay workers, police and justice system	All domains, except macro level	1	0
Home for Socio- psychological Rehabilitation for the Mentally Ill, Thirupattur, India (71–73)	Residential centre, NGO	Residential shelter, reintegration	Outreach teams	Not stated	All domains, except macro level	3	0
Rohtak State Institute of Mental Health, India (74, 75)	Hospital inpatient, governmental	Institutional, reintegration	Admission under judicial reception orders, conveyed by police	MHPs, police and justice system	All domains, except outreach, empowerment, and macro level	2	0
Rural Development Council, India (105)	Residential centre, NGO	Residential shelter, reintegration, long-term group homes	Outreach teams	GPs, GHPs, GHP allied, lay workers	All domains	0	1
Sajida Foundation, Bangladesh (106)	Residential centre, NGO	Residential shelter, reintegration, long-term group homes	Not specified	MHPs, lay workers	All domains	0	1
Schizophrenia Awareness Association, India (107)	Non-residential community-based, NGO	Long-term group homes	Not specified	MHPs, MHP allied, lay workers	All domains, except outreach	0	1
Shekhinah Clinic, Ghana (76, 108)	Residential centre, NGO	Residential shelter	Conveyed by the Ghana Road Transport Union	Lay workers	All domains, except macro level	0	2
SHED, India (109)	Residential centre, NGO	Residential shelter, reintegration	Not specified	Not stated	All domains	0	1
Shraddha Foundation, India (28, 29, 110)	Residential centre, NGO	Residential shelter, reintegration	Outreach teams	MHPs, MHP allied, GPs, GHPs, GHP allied, lay workers	All domains	0	3
Trust Shanthivanam, India (28, 29, 111)	Residential centre, NGO	Residential shelter, reintegration	Outreach teams	GPs, lay workers	All domains	0	3
Udavum Karangal, India (112)	Residential centre, NGO	Residential shelter, reintegration	Outreach teams, conveyed by police or volunteers	MHPs, MHP allied, GPs, GHP allied, police and justice system	All domains, except macro level, All domains	0	1
Udhavum Ullangal, India (71–73, 113)	Residential centre, NGO	Residential shelter, reintegration	Conveyed by police or volunteers	Lay workers, police and justice system	All domains	3	1

CHW=community health worker. GP=general practitioner. GHP=general health professional. MHP=mental health professional. NGO=non-governmental organisation. PEHSMI=people experiencing homelessness who have severe mental illness.

* Reintegration with family; if family reintegration is not possible, reintegration with other NGOs, institutions, etc.

**Table 2:** Evaluations of interventions for people who are homeless and have severe mental illness: qualitative and mixed quantitative-qualitative studies

Study	Study design	Study setting	Sample size	Sex	Diagnosis	Evaluation	Evaluation level	Summary of findings
Qualitative studies								
Antalikova (2020) (35)	Focus group discussions and individual interviews	Myanmar; community	N=20	50% female (n=^10^); 50% male (n=10)	Not stated	Service evaluation of Aung Clinic	PEHSMI	Art and group therapy valuable in creating a safe space for self-expression, fostering acceptance and empowerment; improved emotion regulation through enhanced mental health management; symptom reduction, such as hearing fewer voices or experiencing fewer recurring thoughts
Borysow and Furtado (2014) (45)	Observation and semi- structured interviews	Brazil; multi- sectoral	N=9 (n=4 service users, n=5 workers)	75% female [n=3]; 25% male [n=1] (service users)	Not stated	Assessed factors that influence success and failure of intersectional work	PEHSMI; provider	Location of services hindered access and coordination; fragmented and understaffed health systems unable to meet needs; absence of formal protocols between services limited intersectoral coordination; social assistance services with specialist support crucial for addressing psychosocial needs and accessing health services
Mixed quantitative-qualitative methods studies								
Padmakar et al (2020) (41)	Pre-evaluation and post- evaluation, and qualitative interviews and observation	India; community	N=11	100% female	64% schizophrenia, 27% psychosis, 9% mood disorder with psychotic symptoms	Service evaluation of transition from a hospital setting to a community- based recovery model	PEHSMI; provider	Supported housing associated with significant symptom reduction (p=0·023) on BPRS and significant improvement in physical health (p=0·042) and social relations (p=0·02) subscales of WHOQOL; supported housing associated with positive social and behavioural changes, particularly mobility, participation, and self-care; initial fears and concerns when transitioning from institutional to supported housing; more privacy, less crowding and intrusion of personal space; initial health-care worker challenges overcome with training; difficulties selecting housing facilities
Chaudhury and Ghosh (2014) (63)	Chart review and qualitative interviews, focus group discussions, and informal interaction and observation	India; residential	n=288	100% female	Not stated	Service evaluation of Sarbari	PEHSMI; provider; community; system	45% family reintegration, follow-up for reintegration group (n=131): 73% remained with family, 9% missing from home, 6% referred to government facilities, 4% returned to Sarbari, and 9% unavailable; 42% on regular medication, 8% taking medication irregularly, 19% discontinued medication, 15% not prescribed medication, and 17% unavailable; 15% engaged in supportive employment (ie, biri or papad making) and 7% returned to original work; provides safe, stable, and inclusive living environment; personalised care and supported independence; family reluctance for reintegration due to financial or social constraints and poor knowledge; achievements and ongoing challenges faced by Sarbari shelter and partners (ie, police and government) include obtaining court paperwork and absence of financial support, observed improvements in quality of life and socialisation, and community acceptance; service costs 119 rupees (Rs) per resident per day
Dastoor (2011) (64)	Chart review and qualitative interviews	India; community	n=1114	Not stated	Not stated	Service evaluation of Naya Daur	PEHSMI; provider; community; system	91% provided with food regularly, 69% provided with clothing and hygiene care facilities, and 55% provided with medical treatment; 6% family reintegration; 64% were followed up; 16% with regular treatment; two mental health committees formed (25 people), 87 awareness camps held (2425 people), and 12 advocacy meetings (345 people); community engagement led to improvements in family reintegration, medication access, and reduced community stigma; social workers key care coordinators; establishing rapport was time intensive and encountered barriers (ie, language or distrust); integrated care between partners important to address needs; government services refuse responsibility to provide care to PEHSMI; community care model experiences limitations to handle extreme vulnerability; service costs of 39–150 Rs per day depending on type of treatment
Deste et al (2024) (33)	Pilot controlled trial and qualitative impressions	Togo and Benin; residential	N=36	CRT 22% female (n=4), 78% male (n=14); TAU 28% female (n=5), 72% male (n=13)	Schizophrenia (DSM-5 criteria)	Assessed cognitive performance, psychosocial functioning, and subjective experiences and perspectives	PEHSMI; provider	CRT greater improvements than TAU in processing speed, working memory, verbal memory, cognitive flexibility, and executive function—moderate to large effect sizes; no significant effect between groups for psychosocial functioning; positive feasibility and acceptability feedback from providers and participants

BPRS=Brief Psychiatric Rating Scale. CRT=cognitive remediation therapy. PEHSMI= people experiencing homelessness who have severe mental illness. TAU=treatment as usual. WHOQOL=World Health Organisation quality-of-life scale

**Table 3:** Evaluations of interventions for people who are homeless and have severe mental illness: quantitative studies

Study	Study design	Study setting	Sample size	Sex	Diagnosis	Evaluation	Evaluation level	Summary of findings
Arun et al (2015) (71)	Chart review*	India; institutional	N=112	30% female; 70% male	Common diagnosis was psychosis unspecified	Assessed psychopathology, self-care, communication, social skills, and vocation	PEHSMI	Statistically significant improvement in total PPAC; some improvement was observed in communication, social skills, and occupation; 50% self-care independent within 6 months
Cyrus et al (2020) (70)	Pre evaluation and post evaluation*	Liberia, community	N=96	Not stated	Not stated	Service evaluation of community-based mental health programme	PEHSMI	88% community reintegration: 24% opened businesses, 5% returned to school, and 5% became peer supporters; 13% remained homeless, supported by CHWs
Dasgupta and Chatterjee (2015) (61)	Matched group design	India, residential	N=50 (32% outreach group; 36% shelter group; 32% restoration group)	100% female	Psychotic disorder (F 20–29 according to ICD-10)	Assessed whether living in three different psychosocial programmes has any impact on QOL of people experiencing homelessness and women with psychosis who are reintegrated with family	PEHSMI	Statistically significant (p=0·05) negative correlation between disability and QOL domains social relationships (*r*=−0·329) and environment(*r*=−0·282); QOL psychological health and environment domains for the outreach group differed significantly and in a negative direction from the shelter and restoration group; disability levels significantly affected social relationships in QOL
Dastoor (2011) (62)	Chart review	India; community	N=39	85% female; 15% male	Not stated	Service evaluation of Arogya	PEHSMI; provider	4 9% family reintegration, 21% in psychiatric hospitals, 18% in night shelters, 8% died, and 5% missing; 22 police stations participated; seven hospitals admitted patients
Desai et al (2010) (23)	Chart review	India, multi- sectoral	N=49	31% female; 69% male	69% non- affective psychosis, 10% bipolar affective disorder, 10% severe depressive episode, 8% intellectual disability with behaviour problems, 2% seizure disorder	Service evaluation of Health Outreach Service Model joint initiative by IHBAS, AAA, and DLSA	PEHSMI	Rehabilitation progress: 22% fully achieved, 2% partially achieved, 18% initiated, and 57% not initiated; 14% family reintegration; 8% referred for intensive treatment; 33% regular follow up, 12% irregular follow up, 33% lost to follow up
Eaton (2008) (24)	Cross-sectional	Nigeria; community	N=51	47% female; 53% male	Not stated	Assessed vocational training, when they last saw the psychiatric nurse, and employment status	PEHSMI	36% ex-residents employed; 52% never started work after discharge due to lack of equipment (33%), mental health relapse or deterioration (28%), social factors (18%), physical health deterioration (13%), forgot skills (5%), or refused to work (2·5%)
Gouveia et al (2017) (59)	Pre- intervention and post- intervention cohort	Mozambique; institutional	N=71	7% female (n=5); 93% male (n=66)	65% schizophrenia and other psychoses, 30% mental and be	Assessed mental health status and potential predictors of family reintegration post treatment	PEHSMI	54% family reintegration; lower reintegration for intellectual disability, higher for substance use disorders, and highest for psychosis diagnosis (χ^2^=6·1; p=0·047)
Gowda et al (2017) (67), Gowda et al (2019)(69)	Chart reivew	India; institutional	N=78	54% female (n=42); 46% male (n=36)	Schizophrenia and other psychotic disorders 65%, 31% intellectual disability, 30% comorbid substance use disorder	Assessed clinical outcome, psychosocial interventions used, discharge and follow up; assess challenges faced by state and society in providing care to PEHSMI	PEHSMI	82% improved at discharge on CGI scale; 51% family reintegration, 19% moved to government institution, 22% moved to NGO or rehabilitation centre, and 8% moved to hospital; developmental disability negatively correlated with family reintegration (B=−2·204; p=0·002); clinical improvement positively correlated with family reintegration (B=2·373; p<0·001); first point of contact for people who are HSMI was 41% police, 41% public, and 18% NGOs
Kumar et al (2019) (74)	Chart review	India; institutional	N=46	40% female (n=19); 60% male (n=27)	85% psychosis not specified, 9% epileptic, 7% intellectual disability	Assessed process of de- institutionalisation and reintegration of PEHSMI	PEHSMI	67% community reintegration; 50% family reintegration; 17% moved to other institution; 30% remained for treatment
Mukherjee et al (2015) (72)	Chart review*	India; residential	N=112	30% female (n=33); 70% male (n=79)	Common diagnosis was psychosis unspecified	Assessed family reintegration after psychosocial rehabilitation	PEHSMI	43% family reintegration; 21% remained for treatment
Ravan et al (2010) (73)	Chart review*	India; residential	Not stated	Not stated	Not stated	Assessed care provided, clinical improvement, rehabilitation, and cost-effectiveness	PEHSMI; system	Clinically significant 2-year improvement in self-care, social skills, insight, and vocational preparedness; rate of improvement higher in first 6 months than second year; greatest improvements in psychiatric symptom severity; service costs of R3292 per person per month
Singh et al (2016) (55)	Chart review	India; institutional	N=82 (57% HMH, 43% GMC)	44% female (n=36); 56% male (n=46)	24% (n=22) psychosis NOS; 49% (n=45) schizophrenia; 11% (n=10) bipolar mood disorder; 8% (n=7) intellectual disability; 2% (n=2) brief psychotic episode; 5% (n=5) substance related	Assessed sociodemographic, illness history, clinical observation and treatment information, and rehabilitation	PEHSMI	41% family reintegration, 9% moved to HMH, 4% to NGO, 11% to government shelter home, 10% missing, and 27% remained for treatment; similar improvement rates across treatment facilities; most improvement in patient’s conditions in HMH (n=20) was 10-30%, and 70-100% in GMC (n=12)
Tripathi (2012) (77)	Chart review*	India; institutional	N=140	13% female; 87% male	87% SMI (F 20, 25, 28, 29, 30·1, 30·2, and 31 categories of ICD-10); remaining had intellectual disability; comorbid substance use, and medical and skin diseases were common	Assessed PEHSMI admitted through valid legal orders	PEHSMI	75% global improvement rating 2 or less at the time of discharge; 68% family reintegration; 14% remained for treatment; 18% rehabilitated in other institution
Tripathi et al (2013) (65)	Chart review	India; institutional	N=140	17% female; 83% male	31% schizophrenia, 34% other primary psychotic disorders, 44% substance misuse and dependence, 13% bipolar disorder/mania, 2% anxiety NOS, 1% personality disorder; intellectual disabilities present in 39%	Assessed sociodemographic, illness history, clinical observation and treatment information, family tracing information, and relocation	PEHSMI	69% family reintegration with treatment follow-up, 9% moved to long-term institution, 2% living with benevolent families, 3% missing, and 1% died; 14% remained for treatment; 10% had CGI- improvement rating 1 or 2, awaiting relocation

AAA=Aashray Adhikar Abhiyan. CGI=clinical global impression. CHWs=community health workers. CRT=cognitive remediation therapy. DLSA=District Legal Services Authority. GMC=psychiatry units of a government medical college. HMH=government-run hospitals for mental health. IHBAS=Institute of Human Behaviour and Allied Sciences. NGO=non-governmental organisation. NOS=not otherwise specified. PEHSMI= people experiencing homelessness who have severe mental illness. PPAC=periodic psychiatric assessment chart. QOL=quality of life.

* Publication type: abstract.
