# Supplementary material for: Programmes for people who are homeless and have severe mental illness in low-income and middle-income countries: a systematic review
Source: Lancet Psychiatry. Author manuscript; Available in PMC 2026 Jan 1. (PMC7618192; doi:10.1016/S2215-0366(25)00206-8)
Supplement: Supplementary Appendix [file EMS208933-supplement-Supplementary_Appendix.docx]

**Supplementary Appendix**

## Appendix1: Embase search strategy (original)

Embase <1974 to 2023 November 15>

1 exp ill-housed persons/ or Homelessness/ or Homeless person/ or Homeless persons/ or Person, homeless/ or Persons, homeless/ or Street people/ or People, street/ or indigent/ or Vagrant/ or Person, vagrant/ or Squat/ or unhoused persons/ or unhoused person/ or persons, unhoused/ or person, unhoused/ or shelterless person/ or shelterless persons/ 17743

2 (homeless* or unhoused or unsheltered or shelterless or "rough sleep*" or roofless or dwellinglessness).mp. 21692

3 ((homeless or ill-housed or unhoused or street or destitute or vagabond or vagran* or squat* or roofless or indigent) adj5 (person* or people or m?n or wom?n)).mp. 8311

4 1 or 2 or 3 23752

5 exp Mental disease/ or exp mental disorder/ or exp Schizophrenia/ or exp Psychosis/ or exp psychotic disorder/ or exp Bipolar disorder/ or Affective disorder, psychotic/ or exp Severe depression/ 2669100

6 ((mental or "severe mental" or psychiatric or behaviour?r) adj5 (disorder* or disease* or illness* or health or impair* or disabilit*)).mp. 765780

7 ("Psychosocial disabil*" or "Mental disabil*").mp. 2344

8 dualdiagnos*.mp. 4675

9 ("neurodevelopmental disabil*" or "intellectual disabil*" or "mental retardation" or autis* or dementia or alzheimer* or "cognitive disorder*" or "cognitive impairment").mp. 698287

10 5 or 6 or 7 or 8 or 9 2980000

11 exp developing countries/ 101223

12 ((developing or developed or "middle income" or "low income" or "lower income") and econom*).mp. 133313

13 (low* and ("gdp gross domestic" or "gross national")).mp. 3046

14 (lmic* or lamic* or "lamicountr*" or "low* income countr*" or "low* middle income countr*" or "middle income countr*" or "third world" or "third world countr*" or "transitional countr*").mp. 68857

15 ((low and middle-income countr*) or lmic or lamic or Afghanistan or Albania or Algeria or Angola or Antigua or Barbuda or Argentina or Armenia or Armenian or Aruba or Azerbaijan or Bangladesh or Benin or Byelarus or Byelorussian or Belarus or Belorussian or Belorussia or Belize or Bhutan or Bolivia or Bosnia or Herzegovina or Hercegovina or Botswana or Brazil or Bulgaria or Burkina Faso or Burkina Fasso or Upper Volta or Burundi or Urundi or Cambodia or Khmer Republic or Kampuchea or Cameroon or Cameroons or Cameron or Camerons or Cape Verde or Central African Republic or Chad or Chile or China or Colombia or Comoros or Comoro Islands or Comores or Mayotte or Congo or Zaire or Costa Rica or Cote d Ivoire or Ivory Coast or Croatia or Cuba or Cyprus or Czechoslovakia or Czech Republic or Slovakia or Slovak Republic or Djibouti or French Somaliland or Dominica or Dominican Republic or East Timor or East Timur or Timor Leste or Ecuador or Egypt or El Salvador or Eritrea or Estonia or Ethiopia or Fiji or Gabon or Gabonese Republic or Gambia or Gaza or Georgia Republic or Georgian Republic or Ghana or Gold Coast or Grenada or Guatemala or Guinea or Guam or Guiana or Guyana or Haiti or Honduras or India or Maldives or Indonesia or Iran or Iraq or Jamaica or Jordan or Kazakhstan or Kazakh or Kenya or Kiribati or Korea or Kosovo or Kyrgyzstan or Kirghizia or Kyrgyz or Kirghiz or Kirgizstan or Lao PDR or Laos or Latvia or Lebanon or Lesotho or Basutoland or Liberia or Libya or Lithuania or Macedonia or Madagasca or Malagasy or Malaysia or Malaya or Malay or Sabah or Sarawak or Malawi or Nyasaland or Mali or Marshall Islands or Mauritania or Mauritius or Agalega Islands or Mexico or Micronesia or Middle East or Moldova or Moldovia or Moldovian or Mongolia or Montenegro or Morocco or Ifni or Mozambique or Myanmar or Myanma or Burma or Namibia or Nepal or Netherlands Antilles or New Caledonia or Nicaragua or Niger or Nigeria or Mariana Islands or Oman or Muscat or Pakistan or Palau or Palestine or Panama or Paraguay or Peru or Philippines or Philipines or Phillipines or Phillippines or Romania or Rumania or Roumania or Russia or Russian or Rwanda or Ruanda or Saint Kitts or St Kitts or Nevis or Saint Lucia or St Lucia or Saint Vincent or St Vincent or Grenadines or Samoa or Samoan Islands or Navigator Island or Navigator Islands or Sao Tome or Senegal or Serbia or Montenegro or Seychelles or Sierra Leone or Slovenia or South Africa or Sri Lanka or Ceylon or Solomon Islands or Somalia or Somaliland or South Africa or Sudan or Suriname or Surinam or Swaziland or Syria or Tajikistan or Tadzhikistan or Tadjikistan or Tadzhik or Tanzania or Thailand or Togo or Togolese or Tonga or Trinidad or Tobago or Tunisia or Turkey or Turkmenistan or Turkmen or Uganda or Ukraine or Uruguay or USSR or Soviet Union or Union of Soviet Socialist Republics or Uzbekistan or Uzbek or Vanuatu or New Hebrides or Venezuela or Vietnam or Viet Nam or West Bank or Yemen or Yugoslavia or Zambia or Zimbabwe or Rhodesia).mp. 2688657

16 11 or 12 or 13 or 14 or 15 2824707

17 (intervention or trial or multicent* or study or evaluation or service or components or implementation or model or "pilot study" or pilot or "evaluation study" or "validation study" or care or multi-sectoral or strateg* or training or "clinical trial" or program* or support or treat*).mp. 29334480

18 ("community mental health" or "mental health" or campaign or awareness-raising or anti stigma or psychosocial or psycholog* or "social work" or therap* or counsel* or "social service" or "case management" or recovery or rehabilitation or "community-based rehabilitation" or CBR or "cash transfer" or microfinance or "faith-based care").mp. 12622822

19 (hous* or shelter or resident* or home or "supported living" or "community-based residential").mp. 1124015

20 ("self help" or "peer support" or peer or befriend* or empower* or family-based or livelihood* or employ* or drop-in or "day centre" or "club house" or "recovery college" or vocation* or educat* or "social inclusion" or support*).mp. 5026976

21 ("substance use" or "physical health" or psychological or "medication adherence" or "medication discontinuation").mp. 1000503

22 17 or 18 or 19 or 20 or 21 31949614

23 4 and 10 and 16 and 22 806

## Appendix 2: Embase search strategy (re-run to final analysis)

Embase <1974 to 2024 June 07>

1 exp ill-housed persons/ or Homelessness/ or Homeless person/ or Homeless persons/ or Person, homeless/ or Persons, homeless/ or Street people/ or People, street/ or indigent/ or Vagrant/ or Person, vagrant/ or Squat/ or unhoused persons/ or unhoused person/ or persons, unhoused/ or person, unhoused/ or shelterless person/ or shelterless persons/ 18442

2 (homeless* or unhoused or unsheltered or shelterless or "rough sleep*" or roofless or dwellinglessness).mp. 22592

3 ((homeless or ill-housed or unhoused or street or destitute or vagabond or vagran* or squat* or roofless or indigent) adj5 (person* or people or m?n or wom?n)).mp. 8639

4 1 or 2 or 3 24691

5 exp Mental disease/ or exp mental disorder/ or exp Schizophrenia/ or exp Psychosis/ or exp psychotic disorder/ or exp Bipolar disorder/ or Affective disorder, psychotic/ or exp Severe depression/ 2821520

6 ((mental or "severe mental" or psychiatric or behaviour?r) adj5 (disorder* or disease* or illness* or health or impair* or disabilit*)).mp. 802328

7 ("Psychosocial disabil*" or "Mental disabil*").mp. 2433

8 dualdiagnos*.mp. 5099

9 ("neurodevelopmental disabil*" or "intellectual disabil*" or "mental retardation" or autis* or dementia or alzheimer* or "cognitive disorder*" or "cognitive impairment").mp. 728959

10 5 or 6 or 7 or 8 or 9 3146514

11 exp developing countries/ 102167

12 ((developing or developed or "middle income" or "low income" or "lower income") and econom*).mp. 138888

13 (low* and ("gdp gross domestic" or "gross national")).mp. 3333

14 (lmic* or lamic* or "lamicountr*" or "low* income countr*" or "low* middle income countr*" or "middle income countr*" or "third world" or "third world countr*" or "transitional countr*").mp. 74569

15 ((low and middle-income countr*) or lmic or lamic or Afghanistan or Albania or Algeria or Angola or Antigua or Barbuda or Argentina or Armenia or Armenian or Aruba or Azerbaijan or Bangladesh or Benin or Byelarus or Byelorussian or Belarus or Belorussian or Belorussia or Belize or Bhutan or Bolivia or Bosnia or Herzegovina or Hercegovina or Botswana or Brazil or Bulgaria or Burkina Faso or Burkina Fasso or Upper Volta or Burundi or Urundi or Cambodia or Khmer Republic or Kampuchea or Cameroon or Cameroons or Cameron or Camerons or Cape Verde or Central African Republic or Chad or Chile or China or Colombia or Comoros or Comoro Islands or Comores or Mayotte or Congo or Zaire or Costa Rica or Cote d Ivoire or Ivory Coast or Croatia or Cuba or Cyprus or Czechoslovakia or Czech Republic or Slovakia or Slovak Republic or Djibouti or French Somaliland or Dominica or Dominican Republic or East Timor or East Timur or Timor Leste or Ecuador or Egypt or El Salvador or Eritrea or Estonia or Ethiopia or Fiji or Gabon or Gabonese Republic or Gambia or Gaza or Georgia Republic or Georgian Republic or Ghana or Gold Coast or Grenada or Guatemala or Guinea or Guam or Guiana or Guyana or Haiti or Honduras or India or Maldives or Indonesia or Iran or Iraq or Jamaica or Jordan or Kazakhstan or Kazakh or Kenya or Kiribati or Korea or Kosovo or Kyrgyzstan or Kirghizia or Kyrgyz or Kirghiz or Kirgizstan or Lao PDR or Laos or Latvia or Lebanon or Lesotho or Basutoland or Liberia or Libya or Lithuania or Macedonia or Madagasca or Malagasy or Malaysia or Malaya or Malay or Sabah or Sarawak or Malawi or Nyasaland or Mali or Marshall Islands or Mauritania or Mauritius or Agalega Islands or Mexico or Micronesia or Middle East or Moldova or Moldovia or Moldovian or Mongolia or Montenegro or Morocco or Ifni or Mozambique or Myanmar or Myanma or Burma or Namibia or Nepal or Netherlands Antilles or New Caledonia or Nicaragua or Niger or Nigeria or Mariana Islands or Oman or Muscat or Pakistan or Palau or Palestine or Panama or Paraguay or Peru or Philippines or Philipines or Phillipines or Phillippines or Romania or Rumania or Roumania or Russia or Russian or Rwanda or Ruanda or Saint Kitts or St Kitts or Nevis or Saint Lucia or St Lucia or Saint Vincent or St Vincent or Grenadines or Samoa or Samoan Islands or Navigator Island or Navigator Islands or Sao Tome or Senegal or Serbia or Montenegro or Seychelles or Sierra Leone or Slovenia or South Africa or Sri Lanka or Ceylon or Solomon Islands or Somalia or Somaliland or South Africa or Sudan or Suriname or Surinam or Swaziland or Syria or Tajikistan or Tadzhikistan or Tadjikistan or Tadzhik or Tanzania or Thailand or Togo or Togolese or Tonga or Trinidad or Tobago or Tunisia or Turkey or Turkmenistan or Turkmen or Uganda or Ukraine or Uruguay or USSR or Soviet Union or Union of Soviet Socialist Republics or Uzbekistan or Uzbek or Vanuatu or New Hebrides or Venezuela or Vietnam or Viet Nam or West Bank or Yemen or Yugoslavia or Zambia or Zimbabwe or Rhodesia).mp. 2802852

16 11 or 12 or 13 or 14 or 15 2943370

17 (intervention or trial or multicent* or study or evaluation or service or components or implementation or model or "pilot study" or pilot or "evaluation study" or "validation study" or care or multi-sectoral or strateg* or training or "clinical trial" or program* or support or treat*).mp. 30314476

18 ("community mental health" or "mental health" or campaign or awareness-raising or anti stigma or psychosocial or psycholog* or "social work" or therap* or counsel* or "social service" or "case management" or recovery or rehabilitation or "community-based rehabilitation" or CBR or "cash transfer" or microfinance or "faith-based care").mp. 13101569

19 (hous* or shelter or resident* or home or "supported living" or "community-based residential").mp. 1167648

20 ("self help" or "peer support" or peer or befriend* or empower* or family-based or livelihood* or employ* or drop-in or "day centre" or "club house" or "recovery college" or vocation* or educat* or "social inclusion" or support*).mp. 5248545

21 ("substance use" or "physical health" or psychological or "medication adherence" or "medication discontinuation").mp. 1029842

22 17 or 18 or 19 or 20 or 21 32977364

23 4 and 10 and 16 and 22 832

24 limit 23 to yr="2023 - 2024" 73

## Appendix3: Peer-reviewed studies excluded at full-text screening stage

| Primary reason for exclusion | Study (first author, year) |
| --- | --- |
| Wrong study design (n=13) | Adekeye, 2023 |
|  | Ayano, 2017 |
|  | Brito, 2022 |
|  | Cervantes, 2015 |
|  | Castanos-Cervantes, 2019 |
|  | Sarajilia, 2014 |
|  | Vale, 2020 |
|  | Kim, 2017 |
|  | Lee, 2017 |
|  | Goncalves, 2017 |
|  | Ul Hassan, 2019 |
|  | Bhattacharya, 2021 |
|  | Bhattacharya, 2022 |
| Wrong population (n=19) | Briggs, 2021 |
|  | Carvalho, 2021 |
|  | Da Silva, 2011 |
|  | De Azevedo-Marques, 2022 |
|  | Montenegro, 2023 |
|  | Foster, 2007 |
|  | Hoffmann, 2017 |
|  | Marques, 2015 |
|  | Nath, 2016 |
|  | Nath, 2016 |
|  | Paula, 2018 |
|  | Koehne, 2017 |
|  | Shein-Szydlo, 2016 |
|  | Tiderington, 2020 |
|  | Bain, 2022 |
|  | Crombach, 2014 |
|  | Emmanuelli, 2011 |
|  | Nambiar, 2022 |
|  | Souza, 2011 |
| Wrong setting (n=15) | Blumenthal, 2015 |
|  | Bovell-Ammon, 2020 |
|  | Brown, 2019 |
|  | Brown, 2023 |
|  | Caplan, 2023 |
|  | Coleman, 2003 |
|  | Cristanti, 2017 |
|  | Garl, 2016 |
|  | Glumbíková, 2020 |
|  | González-Casal, 2022 |
|  | Gutman, 2018 |
|  | McHugo, 2004 |
|  | Ng, 2004 |
|  | Zerger, 2014 |
|  | Zhang, 2018 |
| Insufficient details (n=4) | Arunkumar, 2010 |
|  | Auxilia Catherine, 2022 |
|  | Sivakumar, 2023 |
|  | Susser, 2010 |

## Appendix 4: Domain frequency by programme

| **Basic needs** | |
| --- | --- |
| Shelter (n=5) | AAA, CAPS/night shelter, INCENSE, MA, SC |
| Housing (n=14) | Altruist, Ashadeep, Banyan, GDT, GS, INCENSE, IS, JI, MCT, MDF, PIH Liberia, RDC, SAA, SJF |
| Sanitation and washing facilities (n=45) | ALL |
| Food (n=45) | ALL |
| Clothing (n=45) | ALL |
| Protection (n=45) | ALL |
| **Healthcare** | |
| Psychoeducation intervention (n=15) | AAA, AG, AI, Assoc SC, Banyan, BNG, Edawu, GMCH, IPH, IS, Koshish, NIMHANS, Paripurnata, TS, VLM |
| Psychological intervention (n=28) | AAA, AC, AG, AI, Altruist, Ashadeep, Assoc SC, AT, Banyan, Chittadhama, GMCH, GS, IPH, IS, Koshish, KT, MA, MCT, MDF, MSC, NIMHANS, Paripurnata, SAA, SF, SI, TS, UK, VLM |
| Psychosocial intervention (n=26) | AAA, AC, AI, Ashadeep, AT, Banyan, BNG, CAPS/night shelter, GMCH, GS, INCENSE, IPH, IS, Koshish, KT, MCT, MDF, MSC, NIMHANS, Paripurnata, PIH Liberia, RDC,THPH, TS, UK, UU |
| Provision of medication (n=36) | AAA, AC, AG, AI, Anbagam, Ashadeep, Assoc SC, AT, Banyan, BNG, Chittadhama, Edawu, GDT, GGP, GMCH, GS, ILR, IPH, IS, JI, KGMU, KT, MCT, MDF, MSC, NIMHANS, Paripurnata, PIH Liberia, RSI, SF, SI, THPH, TS, UK, UU, VLM |
| Medication adherence (n=5) | GMCH, ILR, IPH, IS, PIH Liberia |
| Psychiatric diagnosis and assessment (n=38) | AAA, AC, AG, AI, Anbagam, Ashadeep, Assoc SC, AT, Banyan, Chittadhama, Edawu, GDT, GGP, GMCH, GS, ILR, INCENSE, IPH, IS, JI, KGMU, Koshish, KT, MA, MCT, MDF, MSC, NIMHANS, PIH Liberia, RSI, SC, SF, SJF, THPH, TS, UK, UU, VLM |
| Substance-use intervention (n=4) | AAA, BNG, MCT, MDF |
| Physical health/medical intervention (n=25) | AAA, AC, AG, AI, Ashadeep, Assoc SC, AT, Banyan, CAPS/night shelter, Chittadhama, GDT, GMCH, IPH, IS, JI, Koshish, KT, MCT, MDF, NIMHANS, RDC, SC, SI, UK, VLM |
| Occupational therapy/rehabilitation within mental health setting (n=16) | AI, Altruist, Assoc SC, Banyan, CAPS/night shelter, Edawu, IPH, IS, JI, KGMU, KT, MDF, NIMHANS, Paripurnata, THPH, UK |
| **Outreach** | |
| Engagement with people on the streets (n=40) | AAA, AC, AG, AI, Altruist, Anbagam, Ashadeep, Assoc SC, AT, Banyan, BNG, CAPS/night shelter, Chittadhama, Edawu, GDT, GS, ILR, INCENSE, IPH, IS, JI, KGMU, Koshish, KT, MA, MCT, MDF, MSC, NIMHANS, Paripurnata, PIH Liberia, RDC, SJF, SF, SI, THPH, TS, UK, UU, VLM |
| Crisis intervention (n=18) | AAA, AC, AG, Altruist, Ashadeep, AT, Banyan, Chittadhama, GDT, ILR, IS, Koshish, KT, SC, SF, TS, UK, VLM |
| **Service models of care** | |
| Integrated services for people with mental health and substance use conditions (n=6) | AAA, GMCH, IPH, IS, KGMU, NIMHANS |
| Residential/rehabilitation centres (n=27) | AC, AG, AI, Anbagam, Ashadeep, Assoc SC, AT, Banyan, Chittadhama, Edawu, GDT, GS, IS, KT, MA, MCT, MSC, Paripurnata, RDC, SJF, SC, SF, SHED, THPH, TS, UK, UU, VLM |
| Hospital inpatient (n=15) | AAA, AI, Banyan, GGP, GMCH, ILR, IPH, IS, KGMU, Koshish, MDF, NIMHANS, RSI, SF, THPH |
| Hospital outpatient (n=15) | AAA, AI, Ashadeep, Assoc SC, Banyan, GDT, GS, IS, KGMU, KT, MDF, NIMHANS, SC, SF, UK |
| Family-based (n=37) | AAA, AC, AG, AI, Altruist, Anbagam, Ashadeep, Assoc SC, AT, Banyan, BNG, Chittadhama, Edawu, GDT, GMCH, GGP, ILR, INCENSE, IPH, IS, KGMU, Koshish, KT, MA, MCT, MDF, MSC, NIMHANS, Paripurnata, PIH Liberia, RSI, SAA, SF, TS, UK, UU, VLM |
| Individual-based (n=45) | ALL |
| Community-based (n=38) | AAA, AC, AG, AI, Altruist, Anbagam, Ashadeep, Assoc SC, AT, Banyan, BNG, CAPs/night shelter, Chittadhama, Edawu, GDT, GS, INCENSE, IPH, IS, JI, KT, Koshish, MA, MCT, MDF, MSC, Paripurnata, PIH, RDC, SAA, SC, SF, SJF, SI, TS, UK, UU, VLM |
| **Empowerment** | |
| Self-help intervention (n=25) | AAA, AC, AG, AI, Assoc SC, Banyan, BNG, Chittadhama, Edawu, GMCH, GS, GDT, INCENSE, IS, MA, MCT, MSC, Paripurnata, RDC, SAA, SC, SI, THPH, TS, VLM |
| Legal support (n=12) | AAA, AC, AG, Banyan, GS, INCENSE, IS, KGMU, Koshish, KT, RDC, SI |
| Documentation for people with mental illness who are homeless (n=8) | AAA, AG, Banyan, GDT, GGP, IS, KGMU, Koshish |
| Economic support (n=11) | Altruist, Banyan, BNG, GMCH, GS, IS, Koshish, RDC, SC, SF, SI |
| Livelihood opportunities (n=22) | AAA, AG, AI, Altruist, Ashadeep, Assoc SC, AT, Banyan, BNG, Chittadhama, Edawu, GS, INCENSE, IS, Koshish, MDF, Paripurnata, SAA, SC, SF, SI, UK |
| Vocational training (n=28) | AAA, AC, AG, AI, Anbagam, Ashadeep, Assoc SC, AT, Banyan, BNG, Chittadhama, Edawu, INCENSE, IPH, IS, JI, KT, Koshish, MA, MCT, MDF, MSC, Paripurnata, SAA, SI, THPH, TS, UK |
| Social work intervention (n=11) | Banyan, BNG, INCENSE, IPH, IS, Koshish, KT, MA, MCT, PIH Liberia, MDF |
| Integration and inclusion intervention (n=18) | AC, AG, AI, Assoc SC, Banyan, BNG, Chittadhama, Edawu, GS, INCENSE, IS, Koshish, MCT, MDF, Paripurnata, RDC, SAA, SF |
| Recreational therapy (n=15) | AAA, AG, Anbagam, AT, Banyan, GDT, GMCH, KGMU, Koshish, MCT, MSC, Paripurnata, RDC, SAA, TS |
| Religious life (n=6) | AI, AG, Altruist, Banyan, Edawu, MCT |
| **Community level** | |
| Family reintegration (n=40) | AAA, AG, AI, Altruist, Anbagam, Ashadeep, Assoc SC, AT, Banyan, BNG, Chittadhama, Edawu, GDT, GMCH, GGP, GS, ILR, INCENSE, IPH, IS, JI, KGMU, KT, MA, MCT, MDF, MSC, NIMHANS, Paripurnata, PIH Liberia, RDC, RSI, SJF, SI, SF,THPH, TS, UK, UU, VLM |
| Community reintegration/engagement (n=31) | AAA, AC, AG, AI, Altruist, Ashadeep, Assoc SC, Banyan, BNG, Chittadhama, Edawu, GS, INCENSE, IPH, IS, JI, KGMU, Koshish, KT, MCT, MSC, Paripurnata, PIH Liberia, RDC, SAA, SC, SF, TS, UK, UU, VLM |
| Consultation with gate keepers and opinion leaders (n=2) | BNG, GMCH |
| Awareness-raising about mental health and homelessness (n=25) | AAA, AC, AG, AI, Altruist, Ashadeep, Banyan, BNG, Chittadhama, Edawu, GDT, GS, INCENSE, IPH, IS, JI, Koshish, KT, MA, MCT, Paripurnata, RDC, SAA, SF, SI |
| Stigma and discrimination reduction interventions in the community (n=7) | AC, AI, Banyan, BNG, Edawu, GS, IS |
| **Macro level** | |
| Develop national policy on homelessness and/or mental illness (n=3) | Banyan, GS, Koshish |
| Establishing linkages between national, regional and transnational structures (n=25) | AAA, AG, AI, Altruist, Ashadeep, AT, Banyan, Chittadhama, GDT, GS, INCENSE, IS, JI, KGMU, Koshish, KT, MDF, MSC, Paripurnata, RDC, SAA, SF, SI, SJF, TS, UU |
| Introduce mental health indicators relative to homelessness into the national data collection system (n=0) | NONE |
| Programme codes: Aashray Adhikar Abhiyan (AAA); Altruist (altruist); AmaudoItumbauzo(AI); Anbagam (Anbagam); Apna Ghar Ashram (AG); Ashadeep (Ashadeep); Association St Camille (Assoc SC); Atchayam Trust (AT); Aung Clinic (AC); The Banyan (Banyan); BasicNeeds (BNG); CAPS unit and night shelter (CAPS/night shelter); Chittadhama (Chittadhama); Edawu (Edawu); Government medical college and hospital (GMCH); Graminadvasisamajvikassansthan (GS); Green Dot Trust (GDT); Gujarat Government psychiatric care facilities (GGP); INCENSE (INCENSE); Indian Legislation in rehabilitation (ILR); Infulene Psychiatric Hospital (IPH); Iswar Sankalpa (IS); Jewels International (JI); Department of Psychiatry, King George Medical University (KGMU); Koshish (Koshish); Karuna Trust (KT); La Village de L’amour (VLM); Maher Ashram (MA); Mariyasadanam Charitable Trust (MCT); Mendora Foundation (MDF); MS Chellamuthutrust (MSC); Department of Psychiatry at National Institute of Mental Health and Neurosciences (NIMHANS); Paripurnata (Paripurnata); Community-based mental health, Harper and Pleebo districts in Maryland County (PIH Liberia); Home for psychosocial rehabilitation in Thirupattur (THPH); Rohtak State Institute of Mental Health (RSI); Rural Develpoment Council (RDC); Sajida foundation (SJF); Schizophrenia Awareness Association (SAA); Shekhinah Clinic (SC); SHED (SI); Shraddha Foundation (SF); Trust Shanthivanam (TS); UdavumKarangal (UK); UdhavumUllangal (UU) | |

## Appendix5: Risk of Bias

| Table 4. Mixed Methods Appraisal Tool (MMAT) scores per domain and total score, used to assess methodological quality.  Scoring key: Y = Yes, criteria met (1 point); N = No, criteria not met/not possible to assess (0 points); P = criteria partially met (0.5 points); greyed out cell = scoring criteria not applicable | | | | | | | | | | | | | | | | | | | | | | | | | | |
| --- | --- | --- | --- | --- | --- | --- | --- | --- | --- | --- | --- | --- | --- | --- | --- | --- | --- | --- | --- | --- | --- | --- | --- | --- | --- | --- |
|  | 1. Qualitative design | | | | | 2. Quantitative (randomised) design | | | | | 3. Quantitative (non-randomised) design | | | | | 4. Quantitative (descriptive) design | | | | | 5. Mixed-methods design | | | | | MMAT total Score |
| Study ID | 1.1 | 1.2 | 1.3 | 1.4 | 1.5 | 2.1 | 2.2 | 2.3 | 2.4 | 2.5 | 3.1 | 3.2 | 3.3 | 3.4 | 3.5 | 4.1 | 4.2 | 4.3 | 4.4 | 4.5 | 5.1 | 5.2 | 5.3 | 5.4 | 5.5 | % (point) |
| Antalikova(2020) | P | P | P | P | Y |  |  |  |  |  |  |  |  |  |  |  |  |  |  |  |  |  |  |  |  | 60% (3/5) |
| Arun (2015) |  |  |  |  |  |  |  |  |  |  | Y | Y | P | N | N |  |  |  |  |  |  |  |  |  |  | 50% (2.5/5) |
| Borysow& Furtado (2014) | Y | Y | Y | Y | Y |  |  |  |  |  |  |  |  |  |  |  |  |  |  |  |  |  |  |  |  | 100% (5/5) |
| Cyrus (2020) |  |  |  |  |  |  |  |  |  |  |  |  |  |  |  | N | N | Y | Y | N |  |  |  |  |  | 40% (2/5) |
| Dasgupta & Chatterjee (2015) |  |  |  |  |  |  |  |  |  |  | Y | Y | Y | P | Y |  |  |  |  |  |  |  |  |  |  | 95% (4.5/5) |
| Deste (2024) |  |  |  |  |  |  |  |  |  |  | Y | Y | Y | Y | Y |  |  |  |  |  |  |  |  |  |  | 100% (5/5) |
| Eaton (2008) |  |  |  |  |  |  |  |  |  |  |  |  |  |  |  | Y | N | N | N | Y |  |  |  |  |  | 40% (2/5) |
| Gouveia (2017) |  |  |  |  |  |  |  |  |  |  |  |  |  |  |  | Y | N | P | Y | Y |  |  |  |  |  | 70% (3.5/5) |
| Gowda (2017) |  |  |  |  |  |  |  |  |  |  |  |  |  |  |  | N | N | Y | N | Y |  |  |  |  |  | 40%(2/5) |
| Gowda (2019) |  |  |  |  |  |  |  |  |  |  |  |  |  |  |  | N | N | P | N | N |  |  |  |  |  | 10% (0.5/5) |
| Kumar (2019) |  |  |  |  |  |  |  |  |  |  |  |  |  |  |  | N | N | Y | N | N |  |  |  |  |  | 20% (1/5) |
| Mukherjee (2015) |  |  |  |  |  |  |  |  |  |  |  |  |  |  |  | Y | Y | N | N | Y |  |  |  |  |  | 60% (3/5) |
| Padmakae (2020) | Y | Y | P | Y | Y |  |  |  |  |  | P | Y | Y | N | Y |  |  |  |  |  | Y | Y | Y | N | Y | 80% (12/15) |
| Singh (2016) |  |  |  |  |  |  |  |  |  |  | P | P | P | P | Y |  |  |  |  |  |  |  |  |  |  | 60% (3/5) |
| Tripathi (2013) |  |  |  |  |  |  |  |  |  |  |  |  |  |  |  | P | Y | Y | N | P |  |  |  |  |  | 60% (3/5) |
| 1. Qualitative domain questions: Is the qualitative approach appropriate to answer the research question?; 1.2 Are the qualitative data collection methods adequate to address the research question?; 1.3 Are the findings adequately derived from the data?; 1.4 Is the interpretation of results sufficiently substantiated by data?; 1.5 Is there coherence between qualitative data sources, collection, analysis and interpretation? 2. Quantitative (randomised) domain questions: 2.1. Is randomization appropriately performed? 2.2. Are the groups comparable at baseline? 2.3. Are there complete outcome data? 2.4. Are outcome assessors blinded to the intervention provided? 2.5 Did the participants adhere to the assigned intervention? 3. Quantitative (non-randomised) domain questions: 3.1 Are the participants representative of the target population? 3.2 Are measurements appropriate regarding bout the outcome and the intervention (or exposure)? 3.3 Are there complete outcome data? 3.4 Are the confounders accounted for in the design and analysis? 3.5 During the study period, is the intervention administered (or exposure occurred) as intended? 4. Mixed-methods domain questions: 4.1 Is there an adequate rationale for using a mixed methods design to address the research question?; 4.2 Are the different components of the study effectively integrated to answer the research question?; 4.3 Are the outputs of the integration of qualitative and quantitative components adequately interpreted?; 4.4 Are divergences and inconsistencies between quantitative and qualitative results adequately addressed?; 4.5 Do the difference components of the study adhere to the quality criteria of each tradition of the methods involved? | | | | | | | | | | | | | | | | | | | | | | | | | | |

## Appendix6: Domain and related programmecomponents

| **Domain** | **Domain definition** | **Programme components within each domain** |
| --- | --- | --- |
| Basic needs | The *absolute minimum resources necessary for long-term physical well-being* and includes food and water, clothing, and physical safety, including shelter and access to sanitation. Health is also a basic human need but will be considered separately in the second domain | Shelter, housing, sanitation and washing facilities, food, clothing, protection |
| Healthcare | The efforts made to restore and maintain a person’s physical and/or mental health via the diagnosis and treatment of physical and mental illnesses or other impairments | Psychoeducation, psychological intervention, psychosocial intervention, provision of psychotropic medication, psychiatric diagnosis and assessment, substance-use intervention, physical health/medical intervention, occupational therapy/rehabilitation within mental health setting |
| Outreach/engagement | The work done with people in the areas where they are sleeping rough | Engagement with people on the streets, crisis intervention |
| Service models of care | Broadly describe the way health services are delivered, as the best practices for care and service provision for a person, population group or patient cohort as they are supported through their health and social needs | Integrated services for people with mental health and substance use conditions, residential/rehabilitation centres, hospital inpatient, hospital outpatient, family-based, individual-based, community-based |
| Empowerment | Includes activities which increase the level of choice, influence and control that users of mental health services can exercise over events in their lives. Recovery is defined in terms of the persons own values and life goals, and does not necessarily mean reduction of symptoms of mental illness | Self-help intervention, legal support, documentation for people with mental illness who are homeless, economic support, livelihood opportunities, vocational training, social work intervention, integration and inclusion intervention, recreational therapy, religious life |
| Community level | Programmes and initiatives aimed to improve the health and well-being of specific groups of people within a local community. These interventions are often multicomponent, employing multiple strategies and tactics to achieve their goals | Family reintegration, community reintegration/engagement, consultation with gate keepers and opinion leaders, awareness-raising about mental health and homelessness, stigma and discrimination reduction interventions in the community |
| Macro level | Implementation approaches at a higher/systemic level to enhance the delivery of service | Develop national policy on homelessness and/or mental illness, establishing linkages between national, regional and transnational structures, introduce mental health indicators relative to homelessness into the national data collection system |

## Appendix7: Expert Recommendations for Implementing Change (ERIC) Discrete Implementation Strategy Compilation

| **Strategy** | **Definitions** |
| --- | --- |
| 1. **Access new funding** | Access new or existing money to facilitate the implementation |
| 1. **Alter incentive/allowance structures** | Work to incentivize the adoption and implementation of the clinical innovation |
| 1. **Alter patient/consumer fees** | Create fee structures where patients/consumers pay less for preferred treatments (the clinical innovation) and more for less-preferred treatments |
| 1. **Assess for readiness and identify barriers and facilitators** | Assess various aspects of an organization to determine its degree of readiness to implement, barriers that may impede implementation, and strengths that can be used in the implementation effort |
| 1. **Audit and provide feedback** | Collect and summarize clinical performance data over a specified time period and give it to clinicians and administrators to monitor, evaluate, and modify provider behavior |
| 1. **Build a coalition** | Recruit and cultivate relationships with partners in the implementation effort |
| 1. **Capture and share local knowledge** | Capture local knowledge from implementation sites on how implementers and clinicians made something work in their setting and then share it with other sites |
| 1. **Centralize technical assistance** | Develop and use a centralized system to deliver technical assistance focused on implementation issues |
| 1. **Change accreditation or membership requirements** | Strive to alter accreditation standards so that they require or encourage use of the clinical innovation. Work to alter membership organization requirements so that those who want to affiliate with the organization are encouraged or required to use the clinical innovation |
| 1. **Change liability laws** | Participate in liability reform efforts that make clinicians more willing to deliver the clinical innovation |
| 1. **Change physical structure and equipment** | Evaluate current configurations and adapt, as needed, the physical structure and/or equipment (*e.g.*, changing the layout of a room, adding equipment) to best accommodate the targeted innovation |
| 1. **Change record systems** | Change records systems to allow better assessment of implementation or clinical outcomes |
| 1. **Change service sites** | Change the location of clinical service sites to increase access |
| 1. **Conduct cyclical small tests of change** | Implement changes in a cyclical fashion using small tests of change before taking changes system-wide. Tests of change benefit from systematic measurement, and results of the tests of change are studied for insights on how to do better. This process continues serially over time, and refinement is added with each cycle |
| 1. **Conduct educational meetings** | Hold meetings targeted toward different stakeholder groups (*e.g.*, providers, administrators, other organizational stakeholders, and community, patient/consumer, and family stakeholders) to teach them about the clinical innovation |
| 1. **Conduct educational outreach visits** | Have a trained person meet with providers in their practice settings to educate providers about the clinical innovation with the intent of changing the provider’s practice |
| 1. **Conduct local consensus discussions** | Include local providers and other stakeholders in discussions that address whether the chosen problem is important and whether the clinical innovation to address it is appropriate |
| 1. **Conduct local needs assessment** | Collect and analyze data related to the need for the innovation |
| 1. **Conduct ongoing training** | Plan for and conduct training in the clinical innovation in an ongoing way |
| 1. **Create a learning collaborative** | Facilitate the formation of groups of providers or provider organizations and foster a collaborative learning environment to improve implementation of the clinical innovation |
| 1. **Create new clinical teams** | Change who serves on the clinical team, adding different disciplines and different skills to make it more likely that the clinical innovation is delivered (or is more successfully delivered) |
| 1. **Create or change credentialing and/or licensure standards** | Create an organization that certifies clinicians in the innovation or encourage an existing organization to do so. Change governmental professional certification or licensure requirements to include delivering the innovation. Work to alter continuing education requirements to shape professional practice toward the innovation |
| 1. **Develop a formal implementation blueprint** | Develop a formal implementation blueprint that includes all goals and strategies. The blueprint should include the following: 1) aim/purpose of the implementation; 2) scope of the change (*e.g.*, what organizational units are affected); 3) timeframe and milestones; and 4) appropriate performance/progress measures. Use and update this plan to guide the implementation effort over time |
| 1. **Develop academic partnerships** | Partner with a university or academic unit for the purposes of shared training and bringing research skills to an implementation project |
| 1. **Develop an implementation glossary** | Develop and distribute a list of terms describing the innovation, implementation, and stakeholders in the organizational change |
| 1. **Develop and implement tools for quality monitoring** | Develop, test, and introduce into quality-monitoring systems the right input—the appropriate language, protocols, algorithms, standards, and measures (of processes, patient/consumer outcomes, and implementation outcomes) that are often specific to the innovation being implemented |
| 1. **Develop and organize quality monitoring systems** | Develop and organize systems and procedures that monitor clinical processes and/or outcomes for the purpose of quality assurance and improvement |
| 1. **Develop disincentives** | Provide financial disincentives for failure to implement or use the clinical innovations |
| 1. **Develop educational materials** | Develop and format manuals, toolkits, and other supporting materials in ways that make it easier for stakeholders to learn about the innovation and for clinicians to learn how to deliver the clinical innovation |
| 1. **Develop resource sharing agreements** | Develop partnerships with organizations that have resources needed to implement the innovation |
| 1. **Distribute educational materials** | Distribute educational materials (including guidelines, manuals, and toolkits) in person, by mail, and/or electronically |
| 1. **Facilitate relay of clinical data to providers** | Provide as close to real-time data as possible about key measures of process/outcomes using integrated modes/channels of communication in a way that promotes use of the targeted innovation |
| 1. **Facilitation** | A process of interactive problem solving and support that occurs in a context of a recognized need for improvement and a supportive interpersonal relationship |
| 1. **Fund and contract for the clinical innovation** | Governments and other payers of services issue requests for proposals to deliver the innovation, use contracting processes to motivate providers to deliver the clinical innovation, and develop new funding formulas that make it more likely that providers will deliver the innovation |
| 1. **Identify and prepare champions** | Identify and prepare individuals who dedicate themselves to supporting, marketing, and driving through an implementation, overcoming indifference or resistance that the intervention may provoke in an organization |
| 1. **Identify early adopters** | Identify early adopters at the local site to learn from their experiences with the practice innovation |
| 1. **Increase demand** | Attempt to influence the market for the clinical innovation to increase competition intensity and to increase the maturity of the market for the clinical innovation |
| 1. **Inform local opinion leaders** | Inform providers identified by colleagues as opinion leaders or “educationally influential” about the clinical innovation in the hopes that they will influence colleagues to adopt it |
| 1. **Intervene with patients/consumers to enhance uptake and adherence** | Develop strategies with patients to encourage and problem solve around adherence |
| 1. **Involve executive boards** | Involve existing governing structures (*e.g.*, boards of directors, medical staff boards of governance) in the implementation effort, including the review of data on implementation processes |
| 1. **Involve patients/consumers and family members** | Engage or include patients/consumers and families in the implementation effort |
| 1. **Make billing easier** | Make it easier to bill for the clinical innovation |
| 1. **Make training dynamic** | Vary the information delivery methods to cater to different learning styles and work contexts, and shape the training in the innovation to be interactive |
| 1. **Mandate change** | Have leadership declare the priority of the innovation and their determination to have it implemented |
| 1. **Model and simulate change** | Model or simulate the change that will be implemented prior to implementation |
| 1. **Obtain and use patients/consumers and family feedback** | Develop strategies to increase patient/consumer and family feedback on the implementation effort |
| 1. **Obtain formal commitments** | Obtain written commitments from key partners that state what they will do to implement the innovation |
| 1. **Organize clinician implementation team meetings** | Develop and support teams of clinicians who are implementing the innovation and give them protected time to reflect on the implementation effort, share lessons learned, and support one another’s learning |
| 1. **Place innovation on fee for service lists/formularies** | Work to place the clinical innovation on lists of actions for which providers can be reimbursed (*e.g.*, a drug is placed on a formulary, a procedure is now reimbursable) |
| 1. **Prepare patients/consumers to be active participants** | Prepare patients/consumers to be active in their care, to ask questions, and specifically to inquire about care guidelines, the evidence behind clinical decisions, or about available evidence-supported treatments |
| 1. **Promote adaptability** | Identify the ways a clinical innovation can be tailored to meet local needs and clarify which elements of the innovation must be maintained to preserve fidelity |
| 1. **Promote network weaving** | Identify and build on existing high-quality working relationships and networks within and outside the organization, organizational units, teams, etc. to promote information sharing, collaborative problem-solving, and a shared vision/goal related to implementing the innovation |
| 1. **Provide clinical supervision** | Provide clinicians with ongoing supervision focusing on the innovation. Provide training for clinical supervisors who will supervise clinicians who provide the innovation |
| 1. **Provide local technical assistance** | Develop and use a system to deliver technical assistance focused on implementation issues using local personnel |
| 1. **Provide ongoing consultation** | Provide ongoing consultation with one or more experts in the strategies used to support implementing the innovation |
| 1. **Purposely reexamine the implementation** | Monitor progress and adjust clinical practices and implementation strategies to continuously improve the quality of care |
| 1. **Recruit, designate, and train for leadership** | Recruit, designate, and train leaders for the change effort |
| 1. **Remind clinicians** | Develop reminder systems designed to help clinicians to recall information and/or prompt them to use the clinical innovation |
| 1. **Revise professional roles** | Shift and revise roles among professionals who provide care, and redesign job characteristics |
| 1. **Shadow other experts** | Provide ways for key individuals to directly observe experienced people engage with or use the targeted practice change/innovation |
| 1. **Stage implementation scale up** | Phase implementation efforts by starting with small pilots or demonstration projects and gradually move to a system wide rollout |
| 1. **Start a dissemination organization** | Identify or start a separate organization that is responsible for disseminating the clinical innovation. It could be a for-profit or non-profit organization |
| 1. **Tailor strategies** | Tailor the implementation strategies to address barriers and leverage facilitators that were identified through earlier data collection |
| 1. **Use advisory boards and workgroups** | Create and engage a formal group of multiple kinds of stakeholders to provide input and advice on implementation efforts and to elicit recommendations for improvements |
| 1. **Use an implementation advisor** | Seek guidance from experts in implementation |
| 1. **Use capitated payments** | Pay providers or care systems a set amount per patient/consumer for delivering clinical care |
| 1. **Use data experts** | Involve, hire, and/or consult experts to inform management on the use of data generated by implementation efforts |
| 1. **Use data warehousing techniques** | Integrate clinical records across facilities and organizations to facilitate implementation across systems |
| 1. **Use mass media** | Use media to reach large numbers of people to spread the word about the clinical innovation |
| 1. **Use other payment schemes** | Introduce payment approaches (in a catch-all category) |
| 1. **Use train-the-trainer strategies** | Train designated clinicians or organizations to train others in the clinical innovation |
| 1. **Visit other sites** | Visit sites where a similar implementation effort has been considered successful |
| 1. **Work with educational institutions** | Encourage educational institutions to train clinicians in the innovation |
| Powell, B.J., Waltz, T.J., Chinman, M.J. *et al.* A refined compilation of implementation strategies: results from the Expert Recommendations for Implementing Change (ERIC) project. *Implementation Sci* **10**, 21 (2015). https://doi.org/10.1186/s13012-015-0209-1 | |

## Appendix8: ERIC Implementation Strategies by programme

| **Programme** | **ERIC strategies** |
| --- | --- |
| Aashray Adhikar Abhiyan, India | 4, 6, 8, 13, 15, 16, 19, 27, 29, 31, 33, 39, 41, 43, 46, 50, 51, 52, 64, 71 |
| Altruist, India | 6, 15, 52 |
| Amaudo Itumbauzo, Nigeria | 6, 7, 15, 16, 17, 19, 24, 27, 29, 41, 31, 34, 38, 52, 59, 69 |
| Anbagam, India | 6, 13, 27, 41, 46, 50, 51, 52 |
| Apna Ghar Ashram, India | 15, 13 |
| Ashadeep, India | 1, 5, 6, 7, 26, 27, 30, 33 |
| Association St Camillie de Lellis, Côte D’Ivoire, Benin and Togo | 15, 34, 59 |
| Atchayam trust, India | 6, 15, 29, 52 |
| Aung Clinic, Myanmar | 1, 15, 17, 41, 50 |
| The Banyan, India | 1, 4, 5, 6, 7, 13, 15, 16, 17, 19, 21, 23, 24, 26, 29, 30, 32, 33, 34, 35, 36, 38, 39, 40, 41, 45, 46, 47, 50, 51, 52, 53, 54, 55, 56, 57, 59, 61, 63, 64, 71, 72 |
| BasicNeeds, Ghana | 6, 7, 15, 16, 17, 18, 19, 23, 24, 26, 30, 35, 38, 39, 40, 41, 43, 46, 47, 50, 51, 52, 56, 57, 59, 60, 73 |
| CAPS unit (Psychosocial Community Centres) and night shelter, Brazil | 6, 8, 10, 52 |
| Chittadhama, India | 1, 4, 6, 13, 27, 41, 46, 50, 51, 52, 64 |
| Edawu, Nigeria | 6, 13, 15, 54, 71 |
| Government medical College and Hospital, India | 17, 31 |
| Graminadvasisamajvikassansthan, India | 6, 31, 38, 52, 69 |
| Green dot trust, India | 6, 52 |
| Gujarat Government psychiatric care facilities, India | 7, 15, 6, 41 |
| INCENSE (Parivartan Trust), India | 1, 4, 6, 15, 17, 18, 20, 23, 24, 30, 33, 39, 40, 41, 46, 47, 51, 52, 56, 61, 63, 64 |
| Indian Legislation in rehabilitation, India | 6, 15, 52 |
| Infulene Psychiatric Hospital, Mozambique | 6, 39, 52 |
| Iswar Sankalpa, India | 1, 4, 5, 6, 7, 13, 15, 16, 17, 19, 21, 23, 24, 26, 29, 30, 32, 33, 34, 35, 36, 38, 39, 40, 41, 45, 46, 47, 50, 51, 52, 53, 54, 55, 56, 57, 59, 61, 63, 64, 71, 72 |
| Jewels international, India | 6, 52 |
| Department of Psychiatry, King George Medical University, India | 6, 7, 10, 52 |
| Koshish, India | 13, 15 |
| Karuna Trust, India | 13, 15 |
| La Village de L’amour, Cameroom | 21, 41, 50 |
| Maher Ashram, India | 6, 15, 30, 41, 52 |
| Mariyasadanam Charitable Trust, India | 1, 6, 13, 64 |
| Menadora Foundation, India | 6, 52 |
| MS Chellamuthutrust, India | 24, 30, 34, 38, 39, 45, 46, 47, 50, 52, 55, 60, 64, 65, 72 |
| Department of Psychiatry at National Institute of Mental Health and Neurosciences, India | 7, 8, 41, 52 |
| Paripurnata, India | 1, 4, 6, 8, 16, 19, 24, 27, 39, 41, 43, 46, 50, 51, 64, 73 |
| Community-based mental health, Harper and Pleebo districts in Maryland County, Liberia | 6, 8, 34, 41, 52 |
| Home for psychosocial rehabilitation in Thirupattur, India | 6 |
| Rohtak State Institute of Mental Health, India | 6, 13, 15 |
| Rural development council, India | 6, 15, 52, 69 |
| Sajida foundation, Bangladesh | 6, 15, 52, 69 |
| Schizophrenia awareness association, India | 6, 15, 52 |
| Shekhinah Clinic, Ghana | 1, 35 |
| SHED, India | 6, 15, 52 |
| Shraddha Foundation, India | 1, 4, 6, 11, 13, 64 |
| Trust Shanthivanam, India | 13, 40, 52 |
| UdavumKarangal, India | 15 |
| UdhavumUllangal, India | 8, 16, 52 |
